# Chromosome-level genome of a leaf vegetable *Glebionis coronaria* provides insights into the biosynthesis of monoterpenoids contributing to its special aroma

**DOI:** 10.1093/dnares/dsac036

**Published:** 2022-10-05

**Authors:** Sen Wang, Anqi Wang, Hengchao Wang, Fan Jiang, Dong Xu, Wei Fan

**Affiliations:** Guangdong Laboratory for Lingnan Modern Agriculture (Shenzhen Branch), Genome Analysis Laboratory of the Ministry of Agriculture and Rural Affairs, Agricultural Genomics Institute at Shenzhen, Chinese Academy of Agricultural Sciences, Shenzhen, Guangdong, China; Guangdong Laboratory for Lingnan Modern Agriculture (Shenzhen Branch), Genome Analysis Laboratory of the Ministry of Agriculture and Rural Affairs, Agricultural Genomics Institute at Shenzhen, Chinese Academy of Agricultural Sciences, Shenzhen, Guangdong, China; Guangdong Laboratory for Lingnan Modern Agriculture (Shenzhen Branch), Genome Analysis Laboratory of the Ministry of Agriculture and Rural Affairs, Agricultural Genomics Institute at Shenzhen, Chinese Academy of Agricultural Sciences, Shenzhen, Guangdong, China; Guangdong Laboratory for Lingnan Modern Agriculture (Shenzhen Branch), Genome Analysis Laboratory of the Ministry of Agriculture and Rural Affairs, Agricultural Genomics Institute at Shenzhen, Chinese Academy of Agricultural Sciences, Shenzhen, Guangdong, China; Guangdong Laboratory for Lingnan Modern Agriculture (Shenzhen Branch), Genome Analysis Laboratory of the Ministry of Agriculture and Rural Affairs, Agricultural Genomics Institute at Shenzhen, Chinese Academy of Agricultural Sciences, Shenzhen, Guangdong, China; Guangdong Laboratory for Lingnan Modern Agriculture (Shenzhen Branch), Genome Analysis Laboratory of the Ministry of Agriculture and Rural Affairs, Agricultural Genomics Institute at Shenzhen, Chinese Academy of Agricultural Sciences, Shenzhen, Guangdong, China

**Keywords:** *Glebionis coronaria*, reference genome, transposable element, vegetable, aroma

## Abstract

*Glebionis coronaria* is a popular vegetable with special aroma and a medical plant in East Asia and Mediterranean, but its biological studies and breeding have been hindered by the lack of reference genome. Here, we present a chromosome-level reference genome of *G. coronaria*, with assembled genome size of 6.8 Gb, which is the largest among all the published genomes of diploid Asteraceae species. The large genome size of *G. coronaria* is mainly caused by the recent widespread explosions of long-terminal-repeat retrotransposons. Analyses of macro-synteny and synonymous mutation rate distribution indicate that the *G. coronaria* genome experienced a whole-genome triplication at 40–45 million years ago, shared with all Asteraceae species. In subtribe Artemisiinae, *Glebionis* arose before the divergence of *Chrysanthemum* from *Artemisia*, and *Glebionis* species evolved much faster than *Chrysanthemum* and *Artemisia* species. In *G. coronaria*, the synthesis genes of monoterpenoids 8-oxocitronellyl enol and isopiperitenone were expanded, and the higher expressions of these expanded genes in leaves and stems may contribute to its special aroma. The *G. coronaria* genomic resources will promote the evolution studies of Asteraceae, the metabolism mechanism studies of bioactive compounds, and the breeding improvement of agronomic traits in *G. coronaria*.

## 1. Introduction

*Glebionis coronaria* is a popular leaf vegetable and also a medical plant in East Asia and Mediterranean.^1^*Glebionis coronaria* is an annual plant propagated by seeds, and its yellow capitulum flowers are similar to the flowers of some *Chrysanthemum* species. Therefore, many previous studies named it as *Chrysnathemum coronarium*,^2^ but recent molecular phylogeny studies propose to place *G. coronaria* in a separate genus *Glebionis*.^3^ As *G. coronaria* contain abundant β-carotene, iron, calcium, and other nutrients and have special pleasant aroma,^4^ the shoots are consumed as vegetable in China, Korea, and Japan. Moreover, the essential oils extracted from *G. coronaria* contain many secondary metabolites such as camphor, pinene, and chrysanthenyl, which have antimicrobial, antioxidant, antiviral, and antimycotic activities.^5^ Thus, *G. coronaria* has also been used as a traditional medicine to treat pain, constipation, and cough in ancient times.^6^

The production of secondary metabolites can help plants adapt to changing environments and promote their survival and reproduction. As a large group of plant secondary metabolites, terpenoids are widespread in various plants and participate in photosynthesis (carotene), membrane fluidity (sterol), hormone metabolism (gibberellin, abscisic acid), etc.^7^ The major component of plant terpenoids is species specific, such as taxol (*Taxus chinensis*) and artemisinin (*Artemisia annua*), contributing to their specific biological characteristics.^8^ The major compounds of essential oils in Asteraceae plants are also terpenoids, especially the monoterpenoids (C10) which are synthesized from two isoprene (C5) units.^9^ Monoterpenoids are often volatile and have specific smell, contributing to the flower scent of many ornamental plants,^10^ and some monoterpenoids like camphor and linalool are widely used in spice and perfume industry.^11^ Many monoterpenoids can be detected in the essential oils of *G. coronaria*, but the genes involved in the synthesis of monoterpenoids are not clear.

*Glebionis coronaria* belongs to the subfamily Asteroideae of the largest family Asteraceae of flower plants. In the past decade, over 20 Asteraceae genomes have been published (Supplementary Fig. S15), including 6 species of subfamily Cichorioideae like *Lactuca sativa*,^12^ 4 species of subfamily Carduoideae like *Cynara cardunculus*,^13^ and 18 species of Asteroideae such as *Erigeron canadensis*,^14^*Helianthus annuus*,^15^ and *A. annua*.^16^ These genomic studies revealed that the ancestor of all Asteraceae species experienced a whole-genome trilication (WGT) at 40–45 million years ago (MYA),^12,15^ the ancestor of Heliantheae alliance experienced an additional whole-genome duplication (WGD) at ~29 MYA,^12^ and the yacon-unique WGD occurred at 5.6–5.8 MYA.^17^ The genome size of *G. coronaria* is estimated to be ~7 Gb by *C*-value,^18^ which is much larger than other Asteraceae diploid species. However, the reason for large genome of *G. coronaria* is not clear. In this study, we present a chromosome-level reference genome of *G. coronaria*, investigate its phylogeny history and genome evolution, and explore the biosynthesis genes of monoterpenoids contributing to its special aroma.

## 2. Materials and methods

### 2.1. Karyotype analysis

A local cultivar named ‘fanji’ of *G. coronaria* widely grown in Hebei, Anhui, Jiangsu, and Guangdong provinces of China was selected for genome sequencing. The seeds of *G. coronaria* were incubated at 25°C in culture dish, and the fresh root tips of seedlings were sampled for karyotype analysis by fluorescence *in situ* hybridization. Chromosome numbers were counted by staining with the fluorescence dye DAPI and hybridization with telomere-specific oligonucleotide probes. Ploidy was determined by hybridization with 18S rDNA and 5S rDNA-specific probes.

### 2.2. Genome sequencing

Young leaves of a 30-day-old plant of *G. coronaria* grown in greenhouse were sampled for genomic DNA extraction by Tiangen Hi-DNAsecure Plant Kit (DP350) according to the provided protocols. The purity and integrity of extracted DNA were checked by agarose gel electrophoresis and Thermo Fisher Nanodrop 2000 ultraviolet spectrophotometer, and the high-quality DNA sample with main fragment length >23,000 bp and A260/280 1.8–2.0 was used for long-read sequencing library preparation. Then, genomic DNA was sheared into 10,000–15,000 bp fragments by Covaris g-TUBEs according to the provided protocols, and converted to SMRT dumbbell libraries using PacBio SMRTbell Express Template Prep Kit 2.0 according to the provided protocols. The prepared SMRT sequencing libraries were then sequenced on PacBio Sequel II with the Circular Consensus Sequencing (CCS) mode. Due to the large genome size of *G. coronaria*, a total of six SMRT libraries were prepared and sequenced.

Fresh young leaves of the same 30-day-old plant of *G. coronaria* used for genomic sequencing were also used for High throughput *in situ* Chromatin conformation capture (Hi-C) sequencing. First, nuclear DNA of sampled young leaves was cross-linked by soaking in formaldehyde solution for 15 min. Second, the cross-linked genomic DNA was also extracted using Tiangen Hi-DNAsecure Plant Kit (DP350) and *in vitro* digested by the restriction endonuclease MboI at motifs GATC. Third, the digested DNA ends were repaired and marked with biotin, and spatially proximal ends were ligated to form circles. Fourth, the circular DNA fragments were purified and sheared into 350 bp inserts via Covaris S220 Focused Ultrasonicator, and the inserts with biotin mark were pull down by Streptavidin Magnetic Beads. Last, the biotin-marked spatially proximal DNA inserts were converted to short-read sequencing library by Truseq DNA Library Prep Kit, and sequenced on Illumian NovaSeq 6000 sequencer in paired-end 150-bp mode.

### 2.3. Transcriptome sequencing

Total RNA of root, stem, and leaf tissues of 60-day-old *G. coronaria* plants were extracted using QIAGEN RNeasy Plant Mini Kit according to the provided protocols. The purity and integrity of extracted RNA sample were checked by agarose gel electrophoresis, Thermo Fisher Nanodrop 2000 ultraviolet spectrophotometer, and Agilent 2100 Bioanalyzer. High-quality RNA samples with RIN >8 and clear 18S rDNA and 25S rDNA bands were used for transcriptome sequencing. The mRNAs in total high-quality RNA were reverse transcribed to cDNAs using NEBNext Single Cell/Low Input cDNA Synthesis & Amplification Module and PacBio Iso-Seq Express Oligo Kit, according to the provided protocols. Then, the cDNA fragments with lengths of 500–6,000 bp were converted to isoform sequencing (Iso-Seq) libraries by PacBio SMRTbell Express Template Prep Kit 2.0. The Iso-Seq libraries of root, stem, and leaf tissues were barcoded and sequenced on PacBio Sequel II sequencer.

### 2.4. Genome assembly

Before genome assembly, the genomic HiFi sequencing reads with average quality <99% were discarded, and the sequenced reads derived from a Hi-C library were also quality filtered to remove adapter contamination, low-quality or ambiguous bases. Then, we used GCE version 1.02^19^ to estimate the genome size, repeat content, and heterozygosity of *G. coronaria* by K-mer analysis (K = 17) of genomic HiFi reads. Because the estimated heterozygosity is high (~0.5%, Supplementary Fig. S2), we used the Hifiasm version v0.16.1 with Hi-C mode^20^ to obtain haplotype-resolved assemblies of diploid *G. coronaria* [haplotype 1 (hap1) and haplotype 2 (hap2)], through combining HiFi reads with Hi-C data. The completeness of hap1 and hap2 contig assemblies was assessed using BUSCO version 5.2.2^21^ with eudicots_odb10 database. The contig set of hap2 assembly having slightly higher contiguity and BUSCO completeness was selected as the reference genome assembly of *G. coronaria* (Supplementary Table S3). Then the organelle and microbe-derived fragments were identified and removed to get the nucleus contigs, by searching against the 403,174 prokaryote and 23,229 organelle genomes downloaded from the NCBI database, using Minimap version 2.20^22^ and the criterion of identity >0.95 and coverage >0.95.

To obtain the nine pseudochromosome scaffolds of *G. coronaria* through proximity ligation data, we mapped the quality-filtered Hi-C sequencing reads to the reference genome to generate the valid Hi-C contact matrixes among contig bins (size 100,000 bp) using HiC-Pro version 3.1.0.^23^ Then, the contigs >1,000,000 bp were assembled into chromosome-level scaffolds based on the Hi-C linkage information among contig ends, using EndHiC version 1.0^24^ in multi-round mode and manual correction of mis-joined scaffolds according to Hi-C heatmap.

### 2.5. Annotation of repeat elements

We conducted a comprehensive identification of tandem repeats (TRs) and transposon elements (TEs) in *G. coronaria* genome. TRF version 4.07^25^ was used to identify TRs in *G. coronaria* genome. TEs in *G. coronaria* genome were classified into three groups and identified through three steps: (i) Intact TEs including long-terminal-repeat retrotransposons (LTR-RTs), DNA transposons, and Helitron transposons were predicted according to their structural characteristics using EDTA version 2.0.0,^26^ and these intact TEs were clustered as a reference TE library for *G. coronaria*; (ii) Homology TEs in the intact TE-masked genome were identified by sequence similarity to the known TEs in intact TE library, Repbase (plant lineage) database version 26.05, and TE protein database, using RepeatMasker version 4.1.2 (http://www.repeatmasker.org); (iii) Denovo TEs were identified in the Intact and Homology TE-masked genome mainly by high copy number, in which a TE library was firstly created using RepeatModeler version 2.0.2^27^ and classified using TERL,^28^ and the classified TE library was used to identify Denovo TEs using RepeatMasker. The identified Intact, Homology, and Denovo TEs were combined into a non-redundant TE annotation of *G. coronaria* genome. The statistics of TEs at class, order, superfamily, and family level were done using in-house scripts, and the insertion time of intact LRT-RTs was estimated by the sequence divergence of LTR pairs using LTR_retriever.^29^ Then, the *G. coronaria* genome sequences were soft masked (uppercase to lowercase) at all TEs with length over 80 bp for gene prediction.

### 2.6. Annotation of protein-coding genes and non-coding RNA genes

Protein-coding genes in the TE-masked *G. coronaria* genome were predicted using Augustus version 3.4.0,^30^ which integrated the supporting evidence from mRNA transcripts and homologue proteins. The species-specific gene training parameters used by Augustus were obtained from BUSCO assessment of *G. coronaria* genome assembly completeness. The transcript supporting hints used by Augustus were generated by aligning the PacBio Iso-Seq full-length transcripts of root, stem, and leaf tissues to genome using GMAP version 2020-10-27,^31^ and converting the alignments with identify and coverage >95% to hints file using Augustus script blat2hints.pl. The homologue protein supporting hints used by Augustus were produced by aligning the proteome of *A. annua*, *C. nankingense*, *E. canadensis*, and *H. annuus* (Supplementary Table S7) to *G. coronaria* genome using Exonerate version 2.2.0,^32^ and converting the best predicted gene structures to hints file using Augustus script exonerate2hints.pl. BUSCO was also used to assess the completeness of *G. coronaria* gene set with eudicots_odb10 database.

Due to the abundant TE content of *G. coronaria* genome, a post-filtering of transposon genes was conducted for the predicted gene set of *G. coronaria*, in which all genes were firstly functionally annotated by searching against the NCBI-NR database using Diamond version 0.8.28,^33^ and the genes with terms ‘retrovirus’, ‘transposon’, ‘copia’, ‘gypsy’, ‘transposae’, ‘gag-pol’, ‘integrase’, etc. were identified as potential transposon genes and removed from the gene set. Thereafter, the functional annotation of the filtered protein-coding gene set of *G. coronaria* was done by searching against NCBI-NR and KEGG databases using Diamond, and the protein domain annotation was done using InterProScan version 5.52-86.^34^ The genes encoding tRNAs and rRNAs were predicted using tRNAScan-SE version 2.0 and RNAmmer version 1.2, respectively.

### 2.7. Phylogeny reconstruction and divergence time estimation

To reconstruct the phylogenetic history of *G. coronaria* in Asteraceae, we firstly identify the orthogroups (including orthologue and recent paralogue genes) of *G. coronaria* and six Asteroideae species *C. seticuspe* (Plant GARDEN CsGojo-0_v1), *A. annua* (Global Pharmacopoeia Genome Database Phase0), *E. canadensis* (NCBI C_canadensis_v1), *S. sonchifolius* (NCBI ASM2352597v1), *S. rebaudiana* (FigShare 15169491.v1), *H. annuus* (NCBI HanXRQr2.0-SUNRISE), one Cichorioideae species *L. sativa* (NCBI Lsat_Salinax_v7), and one outgroup species *Coffea canephora* (NCBI AUK_PRJEB4211_v1) (Supplementary Tables S11 and S12) using OrthoFinder version 2.5.2^35^ with parameters ‘-M msa -A mafft -T fasttree -1 -y’. The rooted species tree was inferred using STAG and STRIDE methods invoked in OrthoFinder, and species divergence time was estimated using the RelTime method in MEGA11^36^ with one calibration of the divergence of Coffea and Asteraceae at 95–106 MYA, which was obtained from TimeTree (www.timetree.org).

### 2.8. Genome polyploidization analysis

WGD events during *G. coronaria* evolution were determined based on the macro-synteny at chromosome-scale as well as the distribution of synonymous mutation rate (Ks) for syntenic genes within and between species. Firstly, the all-vs-all alignments of the proteome sequences of *C. seticuspe*, *A. annua*, *G. coronaria*, *E. canadensis*, and *H. annuus* were generated using Diamond^33^ in the orthogroup finding by OrthoFinder.^35^ Then, the proteome alignments were used as input for MCScanX^37^ to identify the inter- and intra-species syntenic genomic blocks. The type of duplicate genes within species was determined using the duplicate_gene_classifier in MCScanX.^37^ R packages circlize and ggplot2 were used to draw the inter- and intra-species synteny dot plot and circle plot. The Ks values of intra-species paralogue genes located in syntenic blocks with more than five genes, and inter-species reciprocal best orthologue genes located in syntenic blocks with more than five genes, were calculated using KsKs_Calculatror^38^ with the GMYN model. Ks distribution curves were drawn in Microsoft Excel 2016.

### 2.9. Analysis of genes involved in terpenoid synthesis

The terpenoid synthesis genes in *G. coronaria* genome were identified by homology alignment. Firstly, we downloaded the known genes involved in the synthesis pathway of terpenoid backbones (map00900) and monoterpenoids (map00902) from KEGG database. Then, the protein-coding genes of *G. coronaria* were aligned to the downloaded known terpenoid synthesis genes using Diamond version 0.8.28^33^ with parameter settings ‘blastp --more-sensitive --evalue 0.00001’. The *G. coronaria* genes with best alignment identity >80% and coverage >60% were retained as potential genes involved in terpenoid synthesis. Furthermore, the potential terpenoid synthesis genes were checked for the existence of N-terminal domain pfam01397 and metal-binding domain pfam03936 of terpenoid synthases using HMMER version 3.1b2, and the genes having both domains were identified as final terpenoid synthesis genes in *G. coronaria*.

To compare the copy number of terpenoid synthesis genes among *G. coronaria* and other Asteroideae species, the above method was also used to identify the terpenoid synthesis genes in *A. annua*, *C. seticuspe*, *E. canadensis*, *H. annuus*, and *S. rebaudiana*. For the terpenoid synthesis gene families with more members in *G. coronaria*, we also used Muscle version 3.8.31 to conduct multiple sequence alignment of the genes from six Asteroideae species, and used Fasttree version 2.1.11 to construct an unrooted tree to investigate the gene phylogenetic history. Besides, the recently expanded terpenoid synthesis genes were also checked for their expression levels in root, stem, and leaf of *G. coronaria*, by counting the mapped full-length transcripts from these tissues to genes.

## 3. Results

### 3.1. High-quality reference genome and annotation

**Figure 1.**
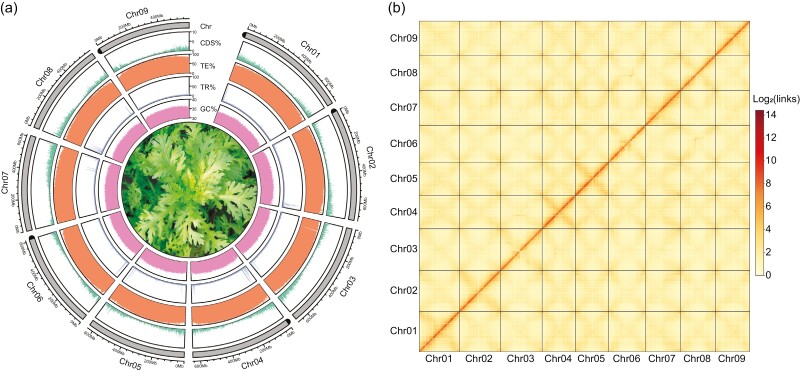
Genome assembly and annotation of *G. coronaria*. (a) Circular view of genomic feature distribution along nine pseudochromosomes. The heights of filled lines in the tracks GC%, TR%, TE%, and CDS% indicate the percent of GC content, accumulated length of TRs, TEs, and gene-coding regions in each 1-Mb sliding window, respectively. In the Chr track, black semicircles at chromosome ends indicate telomeres. (b) Genome-wide Hi-C contact heatmap. Colours are proportional to the Log2-transformed number of Hi-C links in each 3-Mb bin from the same genomic region or between two 3-Mb bins from two different genomic regions.

Karyotype analysis by fluorescence *in situ* hybridization showed that the sequenced material of *G. coronaria* is a diploid with 2*n* = 18 chromosomes (Supplementary Fig. S1), consistent with the previous report.^39^ The estimated genome size was ~6.9 Gb with heterozygosity rate of ~0.5%, using K-mer analysis of 160 Gb PacBio HiFi reads (~23.5×) by GCE^19^ (Supplementary Fig. S2 and Table S1). To resolve the heterozygous problem of *G. coronaria*, the 160 Gb HiFi reads and 100 Gb Hi-C reads (~15.5×) were assembled using Hifiasm^20^ to generate two haplotype-resolved contig assemblies (hap1 and hap2), and the contig set of hap2 assembly with slightly higher BUSCO completeness and contiguity was chosen as the reference genome (Supplementary Table S3). The reference genome of *G. coronaria* includes 5,135 contigs, with N50 size of 3.8 Mb and total length of 6.8 Gb (Table 1, Supplementary Table S3). The assembly size is similar to the estimated genome size, suggesting high completeness of the genome assembly. Then, 85.2% of these contigs were further anchored into nine pseudochromosomes by EndHiC,^24^ with scaffold N50 size of 610.7 Mb (Table 1, Supplementary Tables S2 and S3, Supplementary Fig. S3). Considering that the genome-wide Hi-C contact heatmap had no obvious mistake (Fig. 1b), and the BUSCO completeness of eudicots_odb10 was 94.6% and duplicate rate was 6.4% (Table 1, Supplementary Table S3), the reference genome assembly of *G. coronaria* is in high quality.

**Table 1.** Statistics of genome assembly and annotation of *Glebionis coronaria*

Genomic feature	Value
Estimated genome size (Gb)	6.7
Assembled genome size (bp)	6,799,396,618
Contig N50 size (bp)	3,868,525
Scaffold N50 size (bp)	610,719,037
Anchored to chromosome (%)	85.2%
Telomeres assembled (%)	27.8%
BUSCO completeness of genome	94.6%
Length of TRs (bp)	407,425,333
Percent of TRs (%)	5.9%
Length of TEs (bp)	6,332,052,017
Percent of TEs (%)	93.1%
Number of tRNA genes	8,241
Number of rRNA genes	1,708
Number of protein-coding genes	76,090
Total CDS length (bp)	69,217,854
Percent of total CDS length (%)	1.0%
BUSCO completeness of gene set	94.8%

A comprehensive annotation of repeat elements showed that 5.9% of *G. coronaria* genome were TRs, and 93.1% were TEs (Table 1, Supplementary Table S4). After masking of TEs with length >80 bp, 104,192 gene models were predicted using Augustus^30^ with the supporting evidence of full-length transcript mappings generated by PacBio Isoseq (Supplementary Table S6) and homology protein alignments of well-annotated Asteroideae species (Supplementary Table S7). Of these gene models, 28,102 transposon genes were identified by searching against NCBI-NR database and removed to generate the final 76,090 protein-coding genes, with average CDS length 910 bp and exon number 4.5 per gene (Table 1, Supplementary Table S8). The BUSCO completeness of the gene set was 94.8% and duplicate rate was 8.0% which are comparable to those of the reference genome (Table 1), indicating that both the reference genome and gene set of *G. coronaria* are in high quality and have no obvious heterozygous fragment contamination. In addition, 86.8% of protein-coding genes were functionally annotated by at least one hit from NCBI-NR, KEGG, InterPro, and GO databases (Supplementary Table S10). Furthermore, 8,241 tRNA genes and 1,708 rRNA genes were identified in the genome (Table 1, Supplementary Table S9).

### 3.2. Recent explosion of LTR-RTs results in the large genome of *G. coronaria*

**Figure 2.**
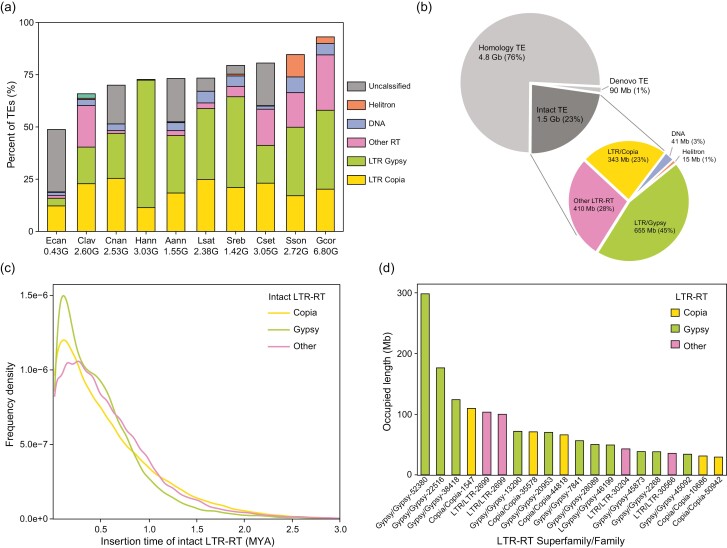
Recent explosion of TEs in *G. coronaria* genome. (a) Accumulated percent of different TE types in the genomes of 10 representative species in subfamily Asteroideae. The TE contents of *E. canadensis* (Ecan), *C. lavandulifolium* (Clav), *C. nankingense* (Cnan), *H. annuus* (Hann), *A. annua* (Aann), *L. sativa* (Lsat), *S. rebaudiana* (Sreb), *C. seticuspe* (Cset), and *S. sonchifolius* (Sson) are obtained from the corresponding genome papers. Numbers below species name indicate genome sizes. (b) Percentages of intact, homology, and Denovo TEs within all TEs in *G. coronaria* genome and the percentages of different TE types within intact TEs. Intact TEs were identified by structural characteristics using EDTA, homology TEs were identified by similarity to known TEs using RepeatMasker, and Denovo TEs were identified by copy number using RepeatModeler. (c) Distribution of the number of intact LTR-RTs along their insertion time in *G. coronaria* genome, estimated by the sequence divergence of LTRs using LTR_retriever. (d) Occupied length of 20 largest LTR-RT families in *G. coronaria* genome.

The genome size of *G. coronaria* is the largest among the published genomes of Asteraceae diploid species, which can be ascribed to the highest TE content up to 93% of *G. coronaria* genome (Fig. 2a). The main TEs in *G. coronaria* genome are LTR-RTs, accounting for 84.2% of genome, and the main LTR-RTs are *Gypsy* and *Copia* superfamily which occupy 37.8% and 20.2% of genome, respectively (Fig. 2a, Supplementary Table S5). The DNA transposon superfamilies Mutator and MC-EnSpm, and Helitron transposons occupy 1.9%, 1.6%, and 3.1% of *G. coronaria* genome, respectively (Supplementary Table S5). Besides, the distribution of TEs is overwhelming along the whole chromosomes (Fig. 1a). Thus, the widespread insertion of LTR-RTs contributes greatly to the large genome of *G. coronaria*.

Further analyses of the TEs in *G. coronaria* genome showed that up to 23% were structurally intact TEs. Within the intact TEs, 45% were *Gypsy* LTR-RTs, 23% were *Copia* LTR-RTs, and 28% were other LTR-RTs (Fig. 2b). In addition, the insertions of these intact LTR-RTs were widespread across the whole chromosomes (Supplementary Fig. S4). Intact TEs were normally inserted recently and still have high activity. In *G. coronaria* genome, most intact LTR-RTs were generated in the past 1 million years, and especially an obvious explosion of *Gypsy* and *Copia* LTR-RTs occurred at ~0.1 MYA (Fig. 2c). The earth at ~0.1 MYA was entering into the Pleistocene ice age, and the explosion of LTR-RTs in *G. coronaria* could create more genetic diversity and novel genes to promote its adaptability to cold environments. In the *G. coronaria* genome, the 20 largest LTR-RT families collectively account for over 25% of TEs, and some *Cypsy* or *Copia* families occupy even more than 100 Mb genomic regions (Fig. 2d, Supplementary Fig. S5). Therefore, the large genome size of *G. coronaria* is mainly caused by the recent explosion of LTR-RTs, and the *G. coronaria* genome may continue increase due to the activity of abundant intact TEs.

### 3.3. *Glebionis* arose before *Chrysanthemum* and evolved fast in Asteroideae

**Figure 3.**
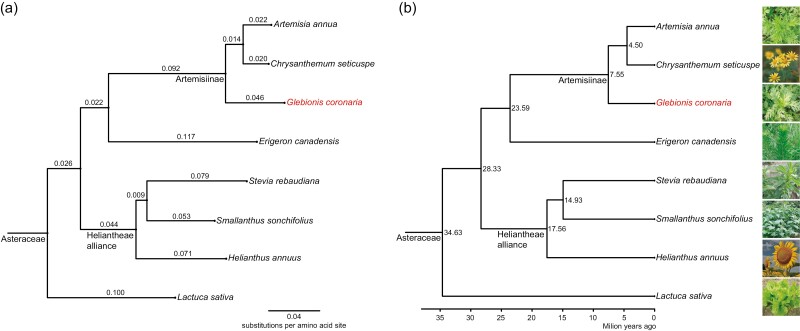
Phylogeny of *G. coronaria* and seven other representative species of Asteraceae. (a) Phylogenetic tree of 8 Asteraceae species built on the gene trees of 2,456 orthogroups with at least 88.9% of species having single-copy genes in any orthogroup, using the STAR and STRIDE methods invoked in OrthoFinder2 with parameters ‘-M msa -A mafft -T fasttree -1 -y’. Float numbers on branches indicate number of substitutions per amino acid site. (b) Time tree of eight Asteraceae species, estimated using RelTime method of MEGA11 with one calibration of the divergence of Coffea and Asteraceae at 95–106 MYA. Float numbers at internodes refer to the estimated species divergence time.

Previous molecular phylogeny studies have moved *G. coronaria* from the genus *Chrysanthemum* to the genus *Glebionis*, which includes only two species *G. coronaria* and *G. segetum*.^40^ Up to now, the detailed phylogeny history of *G. coronaria* is still not clear. To investigate the evolution history of *G. coronaria* in the subfamily Asteroideae, we selected 6 representative Asteroideae species with published genomes, *C. seticuspe*,^41^*A. annua*,^16^*E. canadensis*,^14^*S. sonchifolius*,^17^*S. rebaudiana*,^42^*H. annuus*,^15^ 1 Cichorioideae species *L. sativa*,^12^ and 1 outgroup species *C. canephora*,^43^ and cluster their genes into 37,696 orthogroups using OrthoFinder2^35^ (Supplementary Tables S11 and S12). Then, a rooted phylogeny tree was inferred using STAG and STRIDE methods invoked in OrthoFinder2^35^ based on the gene trees of 2,456 orthogroups with at least 88.9% of species having single-copy genes in any orthogroup (Fig. 3a, Supplementary Fig. S6). Within the subtribe Artemisiinae, *G. coronaria* is a sister to the ancestor of *A. annua* and *C. seticuspe*, suggesting *Glebionis* arose before *Artemisia* and *Chrysanthemum*. In addition, the branch length of *G. coronaria* is much longer than that of *A. annua* and *C. seticuspe* (Fig. 3a), indicating that *G. coronaria* genome has mutated faster and evolved quicker after divergence.

We further estimated the species divergence time in the Asteroideae phylogeny tree (Fig. 3b), using the RelTime method in MEGA11^36^ with one calibration constraint, 95–106 Ma, between coffee and Asteraceae (obtained from TimeTree database). In the estimated time tree, *G. coronaria* diverged from the ancestor of *A. annua* and *C. seticuspe* at 7.55 MYA, and later *A. annua* diverged from *C. seticuspe* at 4.50 MYA (Fig. 3b). The inferred phylogeny history of *G. coronaria* in Asteroideae supports the placement of *G. coronaria* in the genus *Glebionis*, which arose much earlier than the genus *Chrysanthemum*.

### 3.4. Whole-genome triplication of Asteraceae ancestor shapes the genome of *G. coronaria*

**Figure 4.**
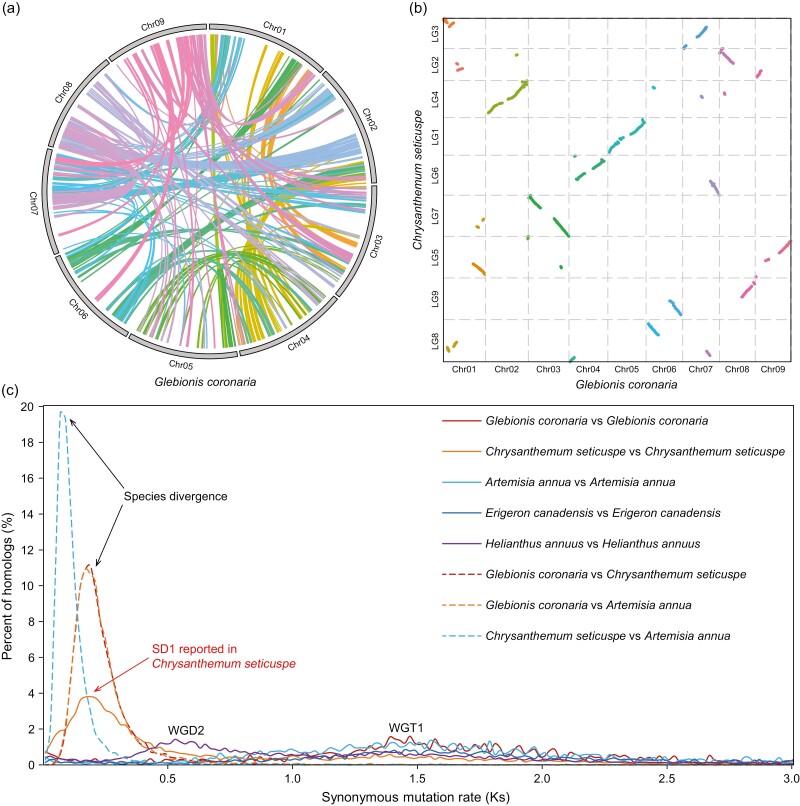
Whole-genome triplication of *G. coronaria*. (a) Circle plot of intra-species synteny blocks in *G. coronaria*, obtained using MCScanX with the all-vs-all alignment of protein-coding genes as input. Each line represents a synteny block with ≥5 genes, and all lines are coloured according to the corresponding chromosome pairs. (b) Dot plot of inter-species synteny between *C. seticuspe* and *G. coronaria*. Each dot represents a synteny block with ≥15 genes, and all dots are coloured according to the corresponding chromosome pairs. (c) Distribution of synonymous mutation rate (Ks) of homologue gene pairs for five Asteroideae species. Ks of intra-species paralogue or inter-species orthologue gene pairs obtained using MCScanX was calculated using KaKs_Calculator with the GMYN model. The previously reported whole-genome triplication (WGT1) for Asteraceae ancestor, whole-genome duplication (WGD2) for the ancestor of Heliantheae alliance, segmental duplication (SD1) for *C. seticuspe*, and the species divergence events are marked on the corresponding Ks peaks.

Genome polyploidization and TE explosion are major driving forces of plant genome evolution, and for the large genome of *G. coronaria* it is necessary to investigate the past genome duplication or triplication events along its evolution history. Because macro-syntenic fragments are important signs of genome duplication, we firstly identified the intra-species syntenic genes in *G. coronaria* and other four Asteroideae species *C. seticuspe*, *A. annua*, *E. canadensis*, and *H. annuus* using MCScanX.^37^ The intra-species synteny plot of *G. coronaria* shows that some genomic blocks are in triplicate (Fig. 4a, Supplementary Fig. S7), for example the syntenic region among the middle part of Chr02, the middle part of Chr07, and the left part of Chr08, indicating the existence of whole-genome triplication during the genome evolution of *G. coronaria*. Besides, the synonymous mutation rate (Ks) distribution of intra-species syntenic genes in *G. coronaria* shows only one peak at ~1.5, similar to other Asteroideae species (Fig. 4c). This Ks peak is corresponding to the widely reported whole-genome triplication (WGT1) event occurred at 40–45 MYA in the ancestor of Asteraceae.^12,15^

In addition to WGT1, the genomes of some *Chrysanthemum* species were reported to undergo recent WGD or segmental duplication (SD) events.^41,44^ For *G. coronaria* and *A. annua*, no sign of recent WGD or SD can be found from intra-species paralogue Ks distribution or inter-species macro-synteny analysis (Fig. 4b and c). Besides, only 7.2% of duplicated genes are predicted to be derived from WGD or SD by MCScanX (Supplementary Table S13), similar to the BUSCO duplicate rate (6.4%) of genome (Supplementary Table S3), both of which indicates no recent WGD event occurred for *G. coronaria*. The nine chromosomes of *G. coronaria* are overall one-to-one to the nine chromosomes of *C. seticuspe* or *A. annua* in the inter-species synteny dot plot (Fig. 4b, Supplementary Figs S8 and S9), indicating no recent WGD occurred in these Asteroideae species. The previously reported SD1 in *C. seticuspe* is also observed at the Ks peak ~0.2. The inter-species Ks peak of *A. annua* vs. *C. seticuspe* is on the left of inter-species Ks peaks of *G. coronaria* vs. *A. annua* and *G. coronaria* vs. *C. seticuspe* (Fig. 4c), indicating that *G. coronaria* arose before the divergence of *A. annua* from *G. coronaria*. This is consistent with the above inferred phylogeny history and divergence time of these three species in the subtribe Artemisiinae (Fig. 3). Therefore, *G. coronaria* did not experience recent WGD after the shared WGT1 event in the ancestor of Asteraceae.

### 3.5. Expansion of 8-oxocitronellyl enol and isopiperitenone synthesis genes contribute to the special aroma of *G. coronaria*

**Figure 5.**
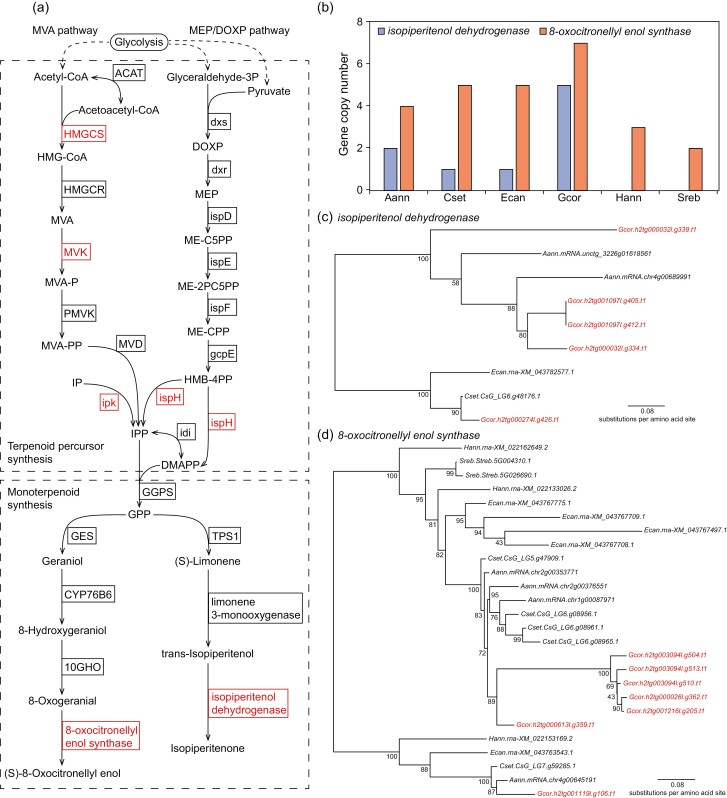
Expansion of terpenoid synthesis genes in *G. coronaria*. (a) Synthesis pathway of terpenoid precursor IPP from MVA and MEP/DOXP, and monoterpenoids 8-oxocitronellyl enol and isopiperitenone. The full names of abbreviated enzymes are given in Supplementary Tables S14 and S15. (b) Gene copy number of *isopiperitenol dehydrogenase* and *8-oxocitronellyl enol synthase* in six Asteroideae species *A. annua* (Aann), *C. seticuspe* (Cset), *E. canadensis* (Ecan), *G. coronaria* (Gcor), *H. annuus* (Hann), and *S. rebaudiana* (Sreb). (c) Phylogeny tree of *isopiperitenol dehydrogenase* gene in Asteroideae species Aann, Cset, Ecan, and Gcor (highlighted in red). (d) Phylogeny tree of *8-oxocitronellyl enol synthase* gene in Asteroideae species Aann, Cset, Ecan, Gcor (highlighted in red), Hann, and Sreb. Phylogeny tree was constructed using FastTree with the multiple protein sequence alignment generated by Muscle. Branch lengths indicate the phylogenetic distances (number of substitutions per amino acid site), and integers at internodes refer to the percent of bootstraps supporting the corresponding splits.

The main aroma compounds in Asteraceae plants are volatile terpenoids, such as monoterpenoids (C10), sesquiterpenoids (C15), and diterpenoids (C20), all of which consist of multiple isoprene (C5) units.^7^ The precursors of terpenoid synthesis in plants, isopentenyl diphosphate (IPP) and dimethylallyl diphosphate (DMAPP), are synthesized through mevalonate (MVA) pathway and methylerythritol phosphate (MEP)/deoxy-xylulose phosphate (DOXP) pathway (Fig. 5a, adapted from KEGG pathway 00900). In the MVA pathway, Acetyl-CoA to is converted to IPP through five consecutive reactions in cytosol.^45^ In the MEP/DOXP pathway, glyceraldehyde phosphate and pyruvate are converted to IPP and DMAPP through seven consecutive reactions in chloroplast.^45^ IPP and DMAPP are further synthesized to geranyl diphosphate (GPP), geranyl geranyl diphosphate (GGPP), famesyl diphosphate (FPP), etc. which can be converted to various monoterpenoids, sesquiterpenoids, and diterpenoids by various terpenoid synthases.^8^

The leaves and stems of *G. coronaria* have special aroma, and the major components of essential oils of *G. coronaria* are monoterpenoids.^1,4^ We identified all the genes involved in the synthesis of monoterpenoids and their precursors IPP and DMAPP, by searching the homologues of the known terpenoid synthesis genes in *G. coronaria* genome. There are totally 65 genes involved in the synthesis of terpenoid precursors (IPP, GPP, FPP, etc.), and 131 genes involved in the synthesis of monoterpenoids (Supplementary Tables S14 and S15) in *G. coronaria*. In particular, the copy numbers of four precursor synthesis genes of MVA pathway (*HMGCS*, *MVK*, *ipk*, and *ispH*) and two monoterpenoid synthesis genes *8-oxocitronellyl enol synthase* and *isopiperitenol dehydrogenase* are much higher in *G. coronaria* than those in the other five Asteroideae species (Fig. 5b, Supplementary Tables S14 and S15). Besides, most copies of these six genes were duplicated after the species divergence of *G. coronaria*, indicating species-specific expansions of these genes (Fig. 5c and d, Supplementary Figs S10–S13). The expansion of these genes may enable *G. coronaria* to produce more terpenoid precursors and monoterpenoids 8-oxocitronellyl enol and isopiperitenone.

Transcriptome sequencing showed that the expression levels of expanded terpenoid synthesis genes in *G. coronaria* were much higher in leaves and stems than those in roots (Supplementary Fig. S14). The major components of the volatile oils extracted from leaves and stems of *G. coronaria* are monoterpenoids geraniol, limonene, and their derivates.^5,46^ The geraniol derivate 8-oxocitronellyl enol and the limonene derivate isopiperitenone have been reported to have special aroma and scent in *Chrysanthemum* plants.^47^ Therefore, the expansion of synthesis genes of 8-oxocitronellyl enol and isopiperitenone may strengthen the synthesis of 8-oxocitronellyl enol and isopiperitenone and contributes to the special aroma of *G. coronaria*.

## 4. Discussion

This study generated a high-quality reference genome and annotation for *G. coronaria*, which has the largest genome size (6.8 Gb) among all the published genomes of diploid Asteraceae species. Our analysis showed the large genome size of *G. coronaria* is mainly caused by the recent explosion of LTR-RTs. Phylogenetic analysis of Asteroideae species supports the current taxonomic placement of *G. coronaria* in *Glebionis* but not *Chrysanthemum*.^40^ In the subtribe Artemisiinae, the genus *Glebionis* arose much earlier (~7.55 MYA) and evolved much faster than the genus *Chrysanthemum* and *Artemisia*. Synteny analysis and Ks distribution indicate that *G. coronaria* genome experienced the WGT1 at 40–45 MYA, shared with all Asteraceae species, and no recent WGD occurred. The *G. coronaria* genomic resources can be used as a model to study the relationship between TE activity and genome evolution, and promote the phylogeny, selection, and evolution studies of Asteraceae.

The special aroma of *G. coronaria* is an important agronomic trait of this vegetable. In the *G. coronaria* genome, we identified a total of 65 genes involved in the synthesis of terpenoid precursors, and 131 genes involved in the synthesis of monoterpenoids. The synthesis genes of monoterpenoids 8-oxocitronellyl enol and isopiperitenone show species-specific expansion in *G. coronaria*. The higher expressions of these expanded genes in leaves and stems of *G. coronaria* may produce more 8-oxocitronellyl enol and isopiperitenone and contribute to the special aroma of *G. coronaria*. Besides, *G. coronaria* also contains abundant unsaturated spiroketal enol compounds, tonghaosu, which has antifeeding activity and protects *G. coronaria* against insects.^48^ Moreover, the essential oils of *G. coronaria* are also used as traditional medicine, due to their antimicrobial, antioxidant, antiviral, and antimycotic activities.^5,49,50^ The generated reference genome here will promote the in-depth studies of the genes involved in the metabolism of these bioactive compounds in *G. coronaria*, the breeding improvement of agronomic traits, and the application of *G. coronaria* in agrochemical and medical industries.

## Supplementary Material

dsac036_suppl_Supplementary_Material

## Data Availability

The genome assembly of *G. coronaria* has been deposited at DDBJ/ENA/GenBank under the accession JANFOE000000000, and the genomic annotation data are deposited at FigShare (10.6084/m9.figshare.20294385). The HiFi reads, Hi-C reads, and full-length transcripts have been deposited in NCBI SRA under the BioProject accession PRJNA851195.
