# Supplementary material for: Chromosome-level genome of a leaf vegetable Glebionis coronaria provides insights into the biosynthesis of monoterpenoids contributing to its special aroma
Source: DNA Res. 2022 Oct 5;29(6):dsac036. doi: 10.1093/dnares/dsac036 (PMC9724771; doi:10.1093/dnares/dsac036)
Supplement: dsac036_suppl_Supplementary_Material [file dsac036_suppl_supplementary_material.docx]

**Supplemental information for:**

**Chromosome-level genome of the leaf vegetable *Glebionis coronaria* provides insights into the biosynthesis of monoterpenoids contributing to its special aroma**

Sen Wang, Anqi Wang, Hengchao Wang, Fan Jiang, Dong Xu, Wei Fan

**Supplemental figures**


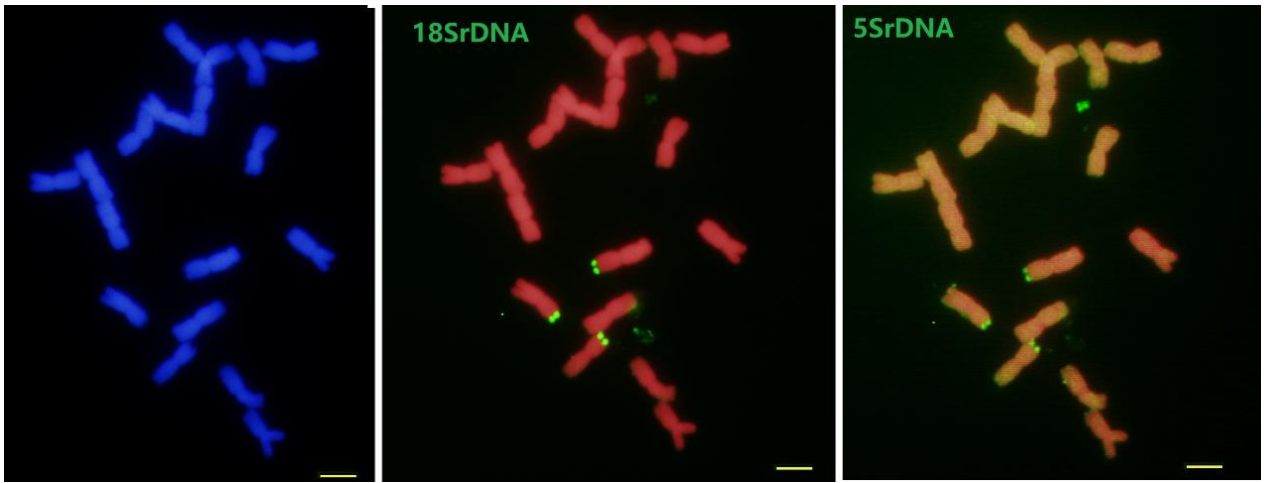


**Figure S1.** Karyotype analysis of *Glebionis coronaria* by fluorescence in situ hybridization (FISH). Left, 18 chromosomes are stained to blue by the fluorescence dye DAPI. Middle, 3 18S rDNA bands (green) are shown by the hybridization with specific probes on the background of red chromosomes. Right, 3 5S rDNA bands (green) are shown by the hybridization with specific probes on the background of orange chromosomes. Bars on the bottom right indicate 5 μm.

**Figure S2.** Distribution of 17-mer frequency in the PacBio HiFi sequencing reads of *Glebionis coronaria*. On the curve, the major peak (black arrow) indicates K-mers from the unique regions of the genome, the peak on the left of the major peak indicates K-mers from the heterozygous regions of the genome, and the peaks on the right of the major peak indicate K-mers from the repetitive regions of the genome. A photograph of Glebionis coronaria is posted on the top right.


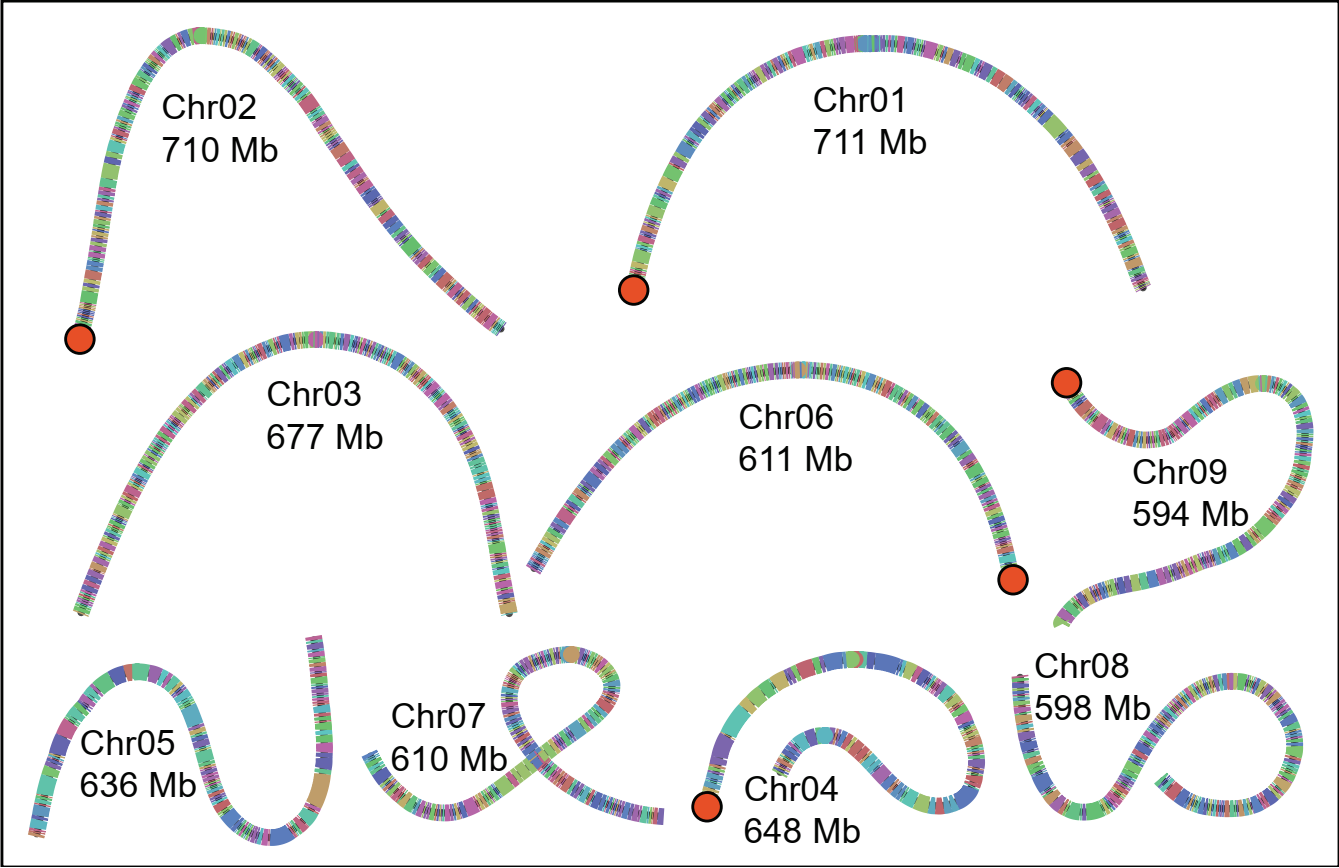


**Figure S3.** Bandage view of 9 chromosome-level scaffolds of *Glebionis coronaria*. The threaded colorful bands indicate the ordered and oriented contigs along chromosomes, and the red solid circles indicate telomeres.


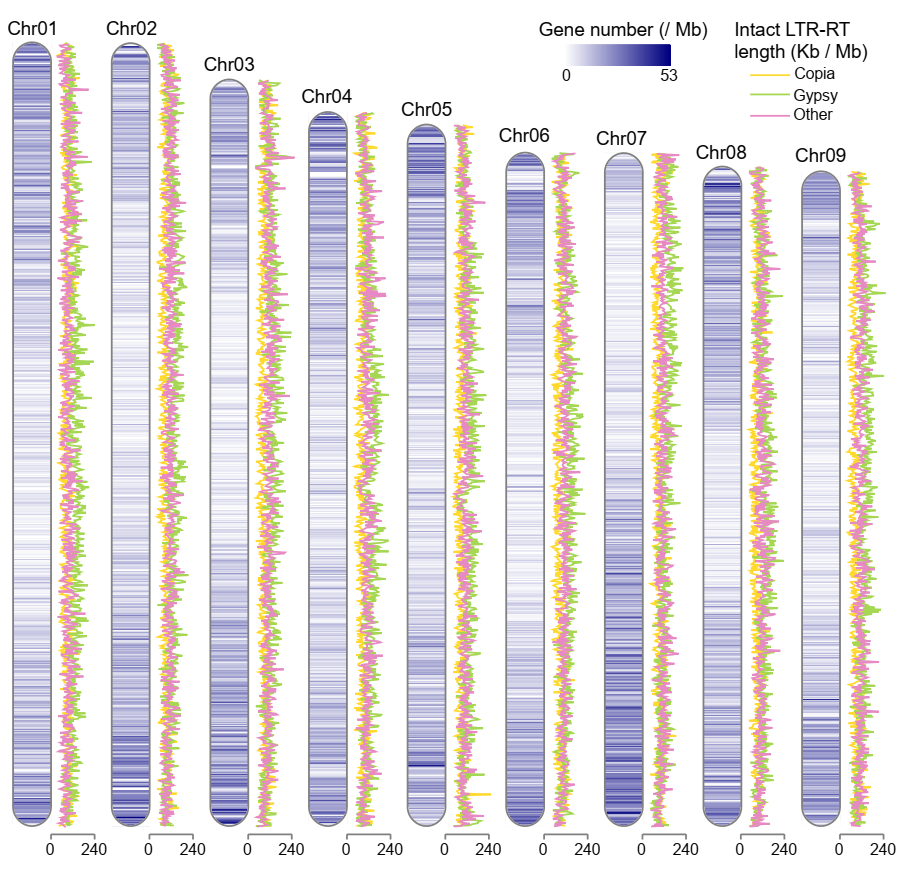


**Figure S4.** Distribution of genes and long terminal repeat retrotransposons (LTR-RTs) along the 9 chromosomes of *Glebionis coronaria*. The darkness of blue bands in chromosomes is proportional to the number of genes in 1-Mb window, the horizontal distances between the solid lines and the right borders of the corresponding chromosomes indicate the occupied length of Copia (yellow line), Gypsy (green line), and Other (pink line) LTR-RTs.


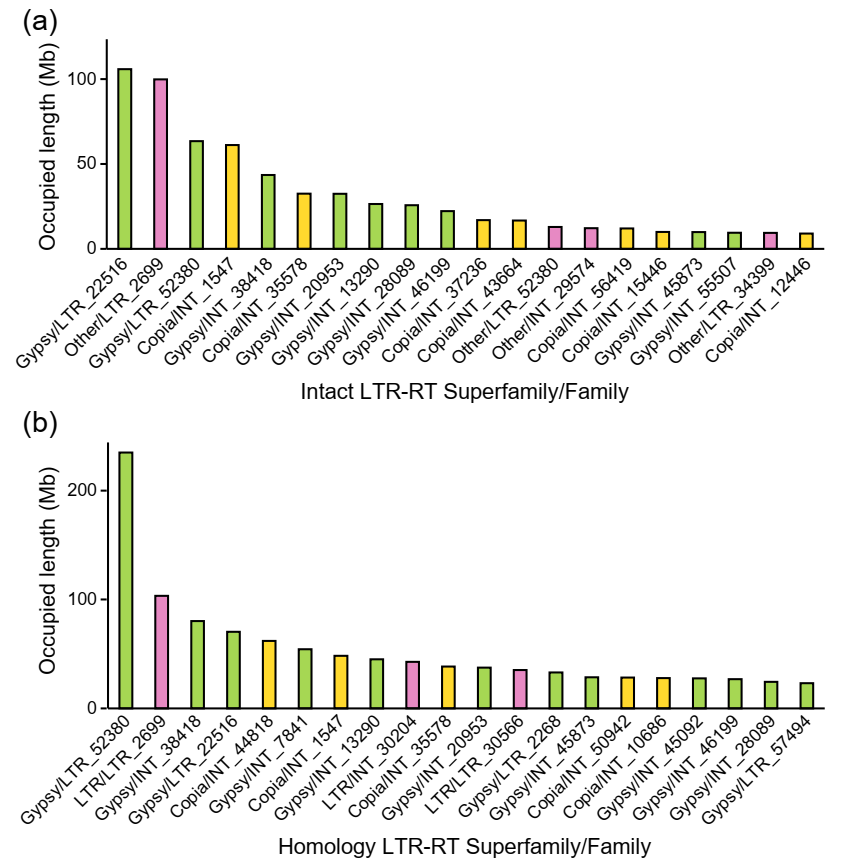


**Figure S5.** Occupied length of the largest 20 families of intact (a) and homology (b) long terminal repeat retrotransposons (LTR-RTs) in the genome of *Glebionis coronaria*. Intact LTR-RTs were identified by their structural characteristics using EDTA, and Homology LTR-RTs were identified by their sequence similarities to known LTR-RTs in Repbase or the intact LTR-RT library built by EDTA.


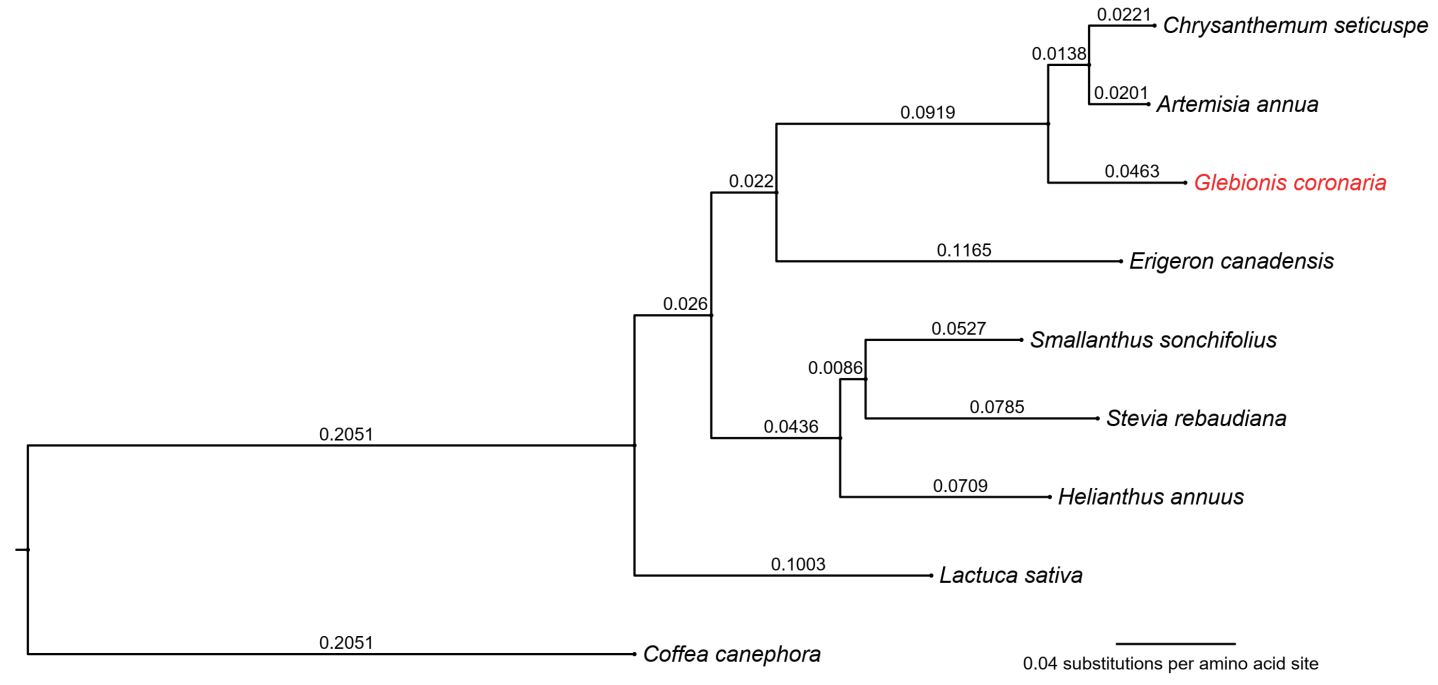


**Figure S6.** Phylogeny tree of 8 Asteraceae species and the outgroup *Coffea canephora*. The tree was constructed based on the concatenated multiple protein sequence alignment of 2,456 orthogroups with at least 88.9% of species having single-copy genes in any orthogroup, which were obtained by OrthoFinder2 with parameters “-M msa -A mafft -T fasttree -1 -y”. Branch lengths refer to the phylogenetic distances as indicated by number of substitutions per amino acid site (float numbers on branches).


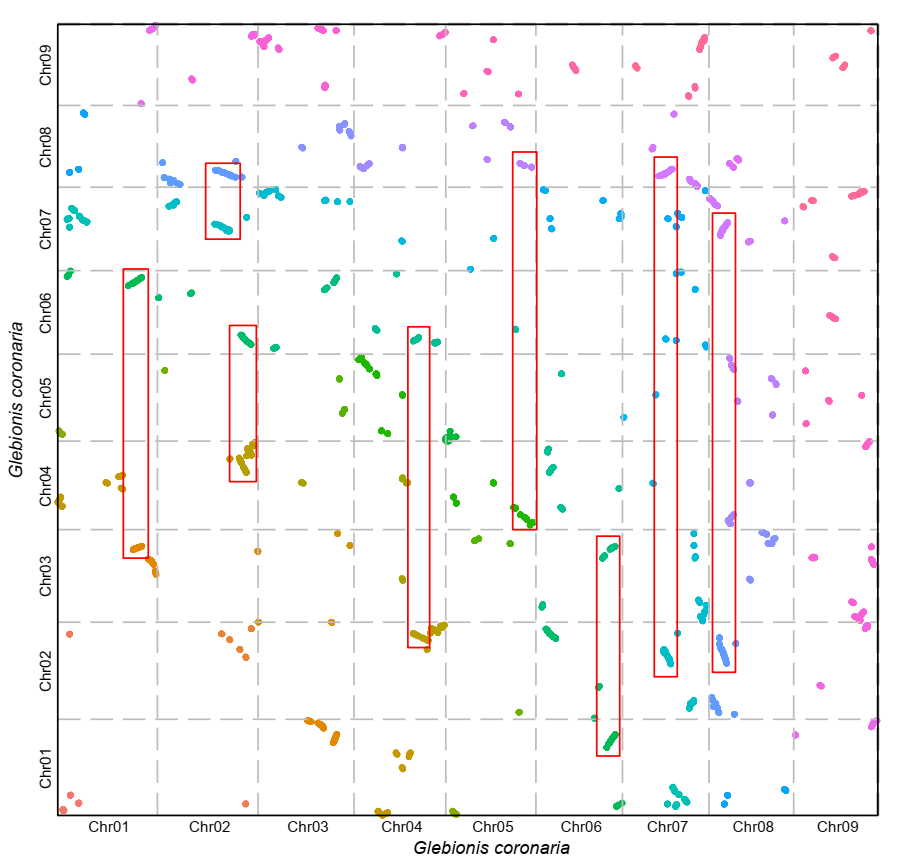


**Figure S7.** Intra-species synteny dot plot of *Glebionis coronaria*. The synteny analysis was done using MCScanX with the all-vs-all alignment of protein-coding genes anchored to chromosomes as input. Each dot represents a synteny block with >= 5 genes, and all dots are colored according to the corresponding chromosome pairs. Red rectangles highlighted the synteny blocks that are in triplicate in *G. coronaria* genome, namely one genomic region of a chromosome (like Chr02) has two corresponding syntenic regions in another two chromosomes (like Chr07 and Chr08). The triplicate of macro synteny indicates the existence of whole genome triplication (WGT) event in the ancestor of Asteraceae.


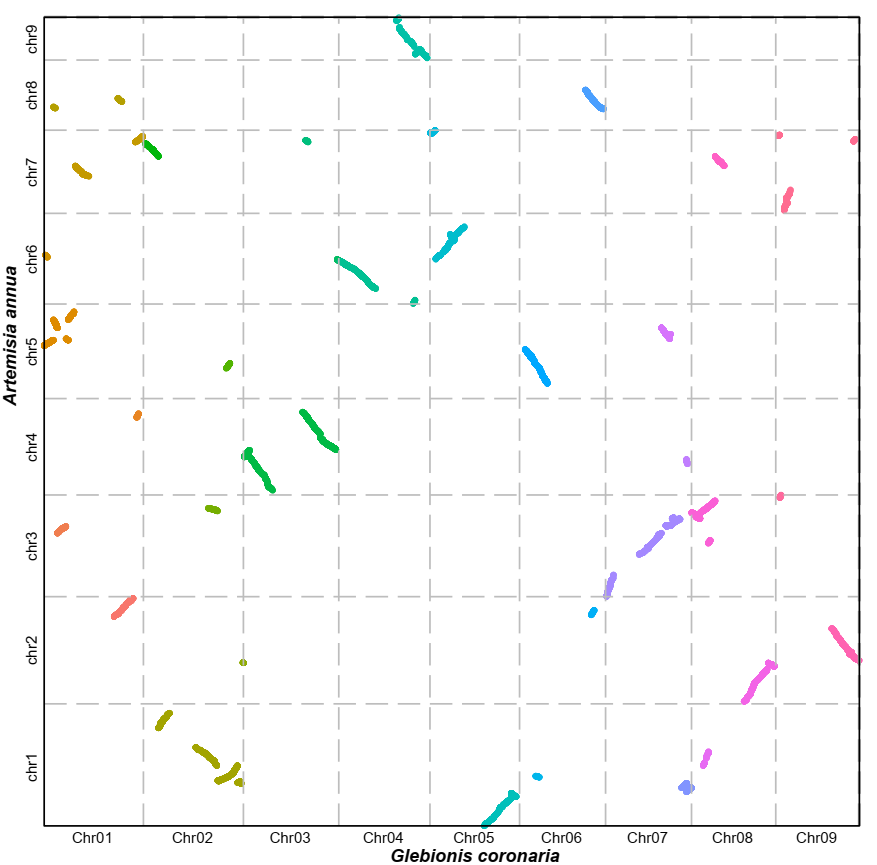


**Figure S8.** Inter-species synteny dot plot of *Artemisia annua* to *Glebionis coronaria*. The synteny analysis was done using MCScanX with the all-vs-all alignment of protein-coding genes anchored to chromosomes as input. Each dot represents a synteny block with >= 15 genes, and all dots are colored according to the corresponding chromosome pairs.


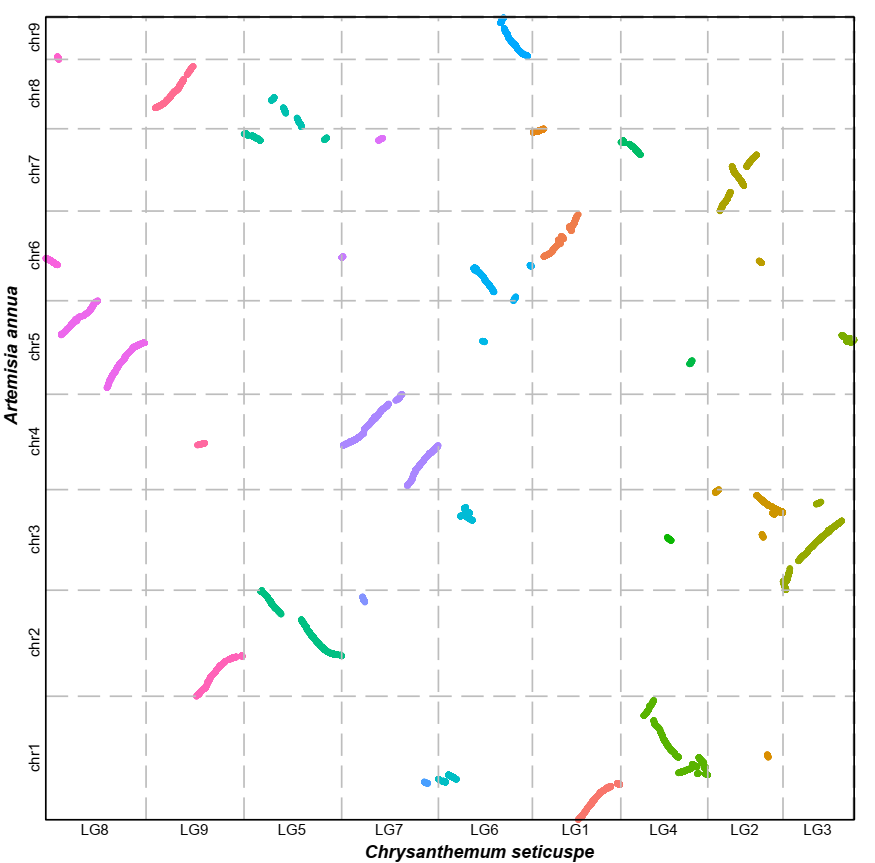


**Figure S9.** Inter-species synteny dot plot of *Artemisia annua* to *Chrysanthemum seticuspe*. The synteny analysis was done using MCScanX with the all-vs-all alignment of protein-coding genes anchored to chromosomes as input. Each dot represents a synteny block with >= 15 genes, and all dots are colored according to the corresponding chromosome pairs.


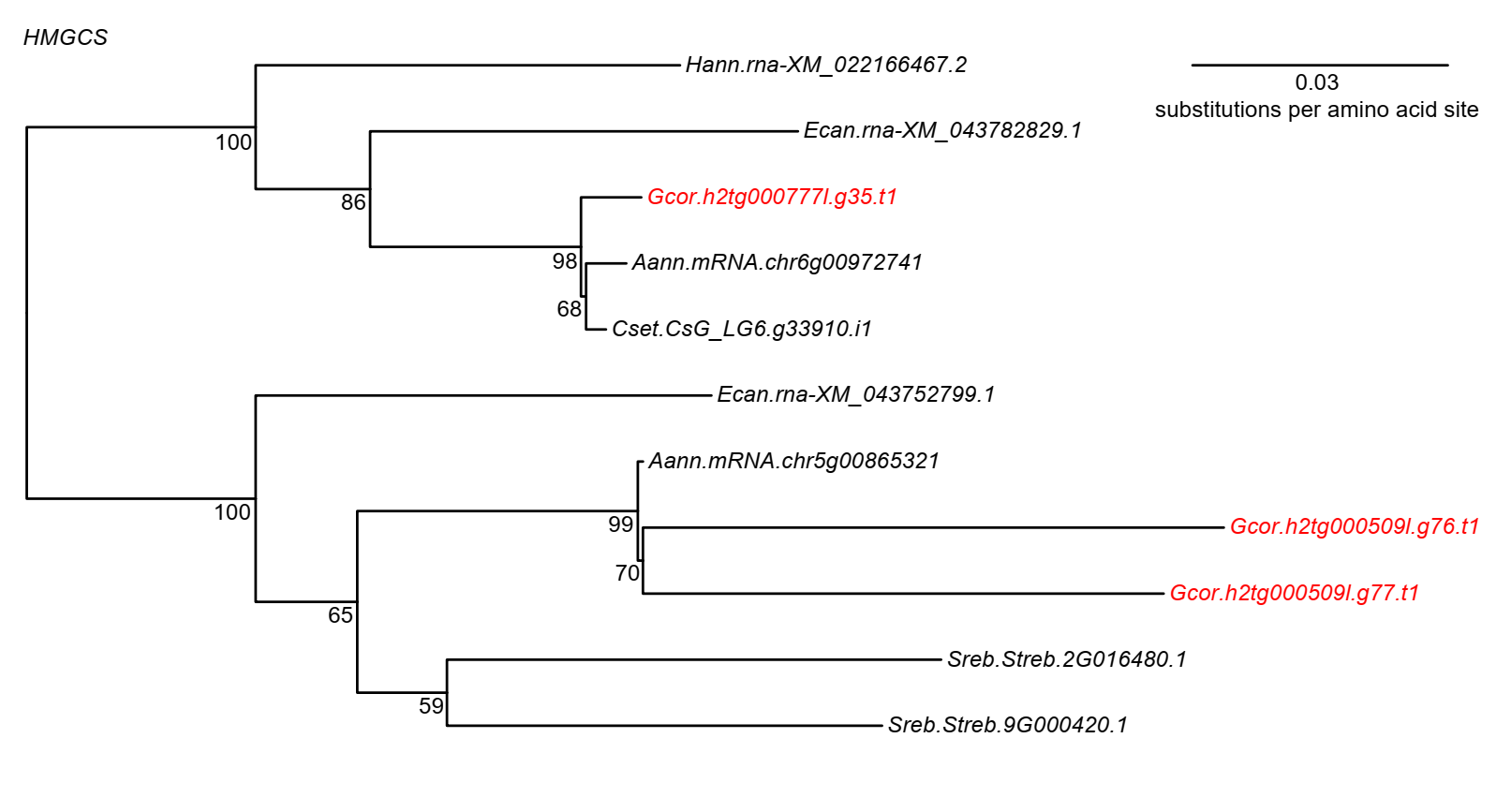


**Figure S10.** Phylogeny tree of hydroxymethylglutaryl-CoA synthase gene (*HMGCS*) in 6 Asteroideae species, *Helianthus annuus (Hann)*, *Stevia rebaudiana (Sreb)*, *Erigeron canadensis (Ecan)*, *Glebionis coronaria (Gcor)*, *Artemisia annua (Aann)*, and *Chrysanthemum seticuspe (Cset)*. The tree was constructed using FastTree version 2.1.11^2^ with the multiple protein sequence alignment generated by Muscle version 3.8.31^3^. Branch lengths indicate the phylogenetic distances and integers at splits refer to the number of bootstraps support the corresponding splits.


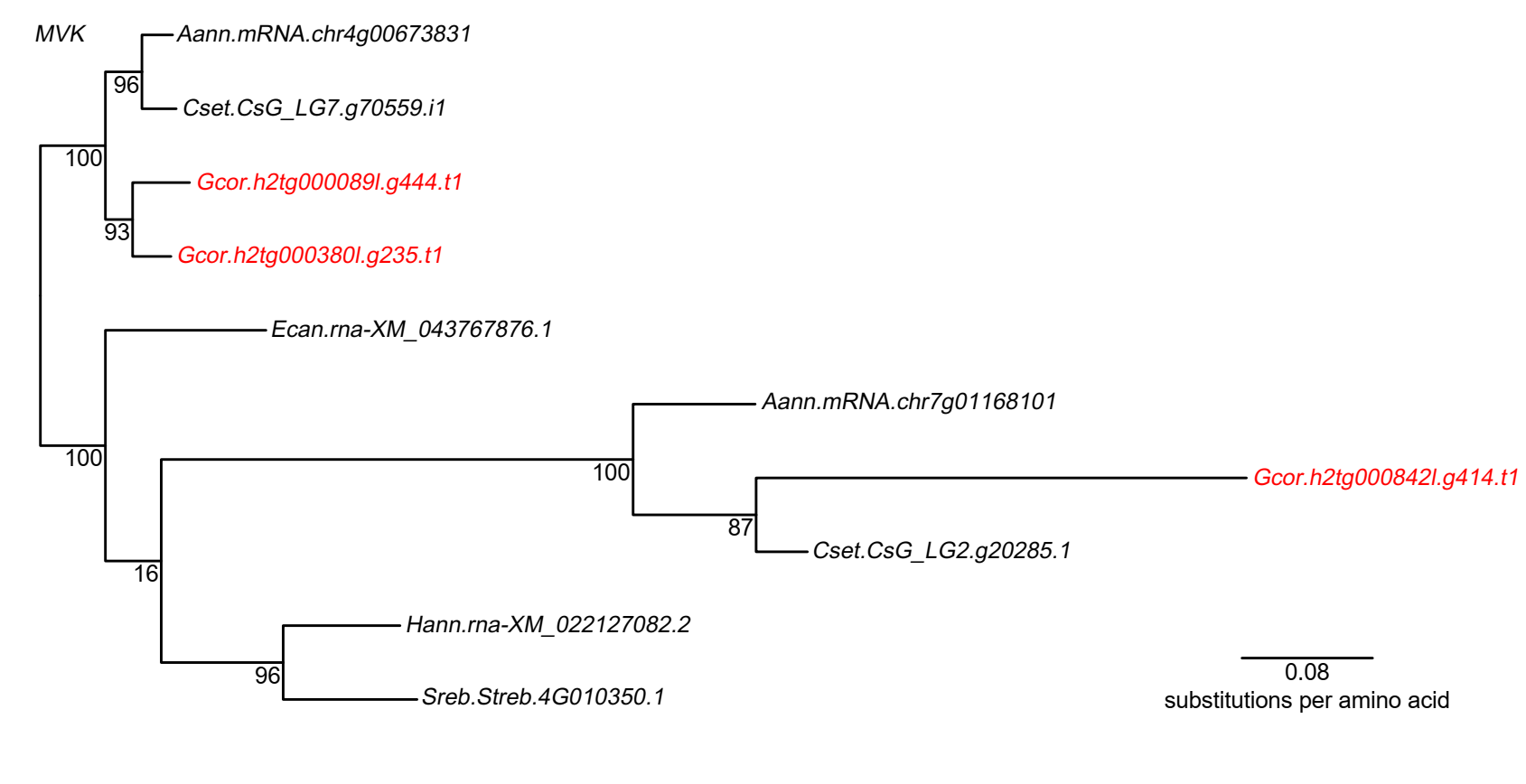


**Figure S11.** Phylogeny tree of mevalonate kinase gene (*MVK*) in 6 Asteroideae species, *Helianthus annuus (Hann)*, *Stevia rebaudiana (Sreb)*, *Erigeron canadensis (Ecan)*, *Glebionis coronaria (Gcor)*, *Artemisia annua (Aann)*, and *Chrysanthemum seticuspe (Cset)*. The tree was constructed using FastTree version 2.1.11^2^ with the multiple protein sequence alignment generated by Muscle version 3.8.31^3^. Branch lengths indicate the phylogenetic distances and integers at splits refer to the number of bootstraps support the corresponding splits.


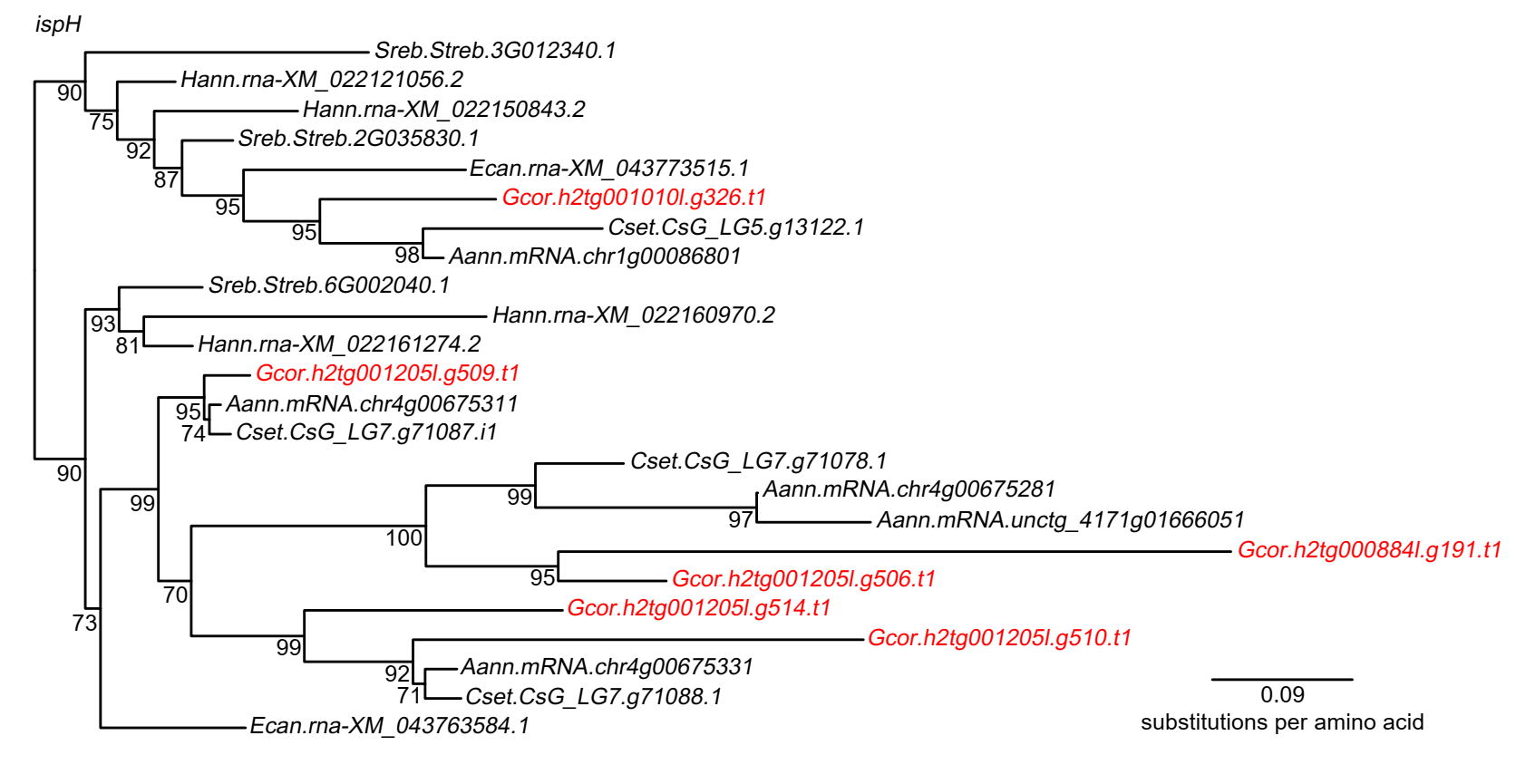


**Figure S12.** Phylogeny tree of 4-hydroxy-3-methylbut-2-enyl diphosphate reductase gene (*ispH*) in 6 Asteroideae species, *Helianthus annuus (Hann)*, *Stevia rebaudiana (Sreb)*, *Erigeron canadensis (Ecan)*, *Glebionis coronaria (Gcor)*, *Artemisia annua (Aann)*, and *Chrysanthemum seticuspe (Cset)*. The tree was constructed using FastTree version 2.1.11^2^ with the multiple protein sequence alignment generated by Muscle version 3.8.31^3^. Branch lengths indicate the phylogenetic distances and integers at splits refer to the number of bootstraps support the corresponding splits.


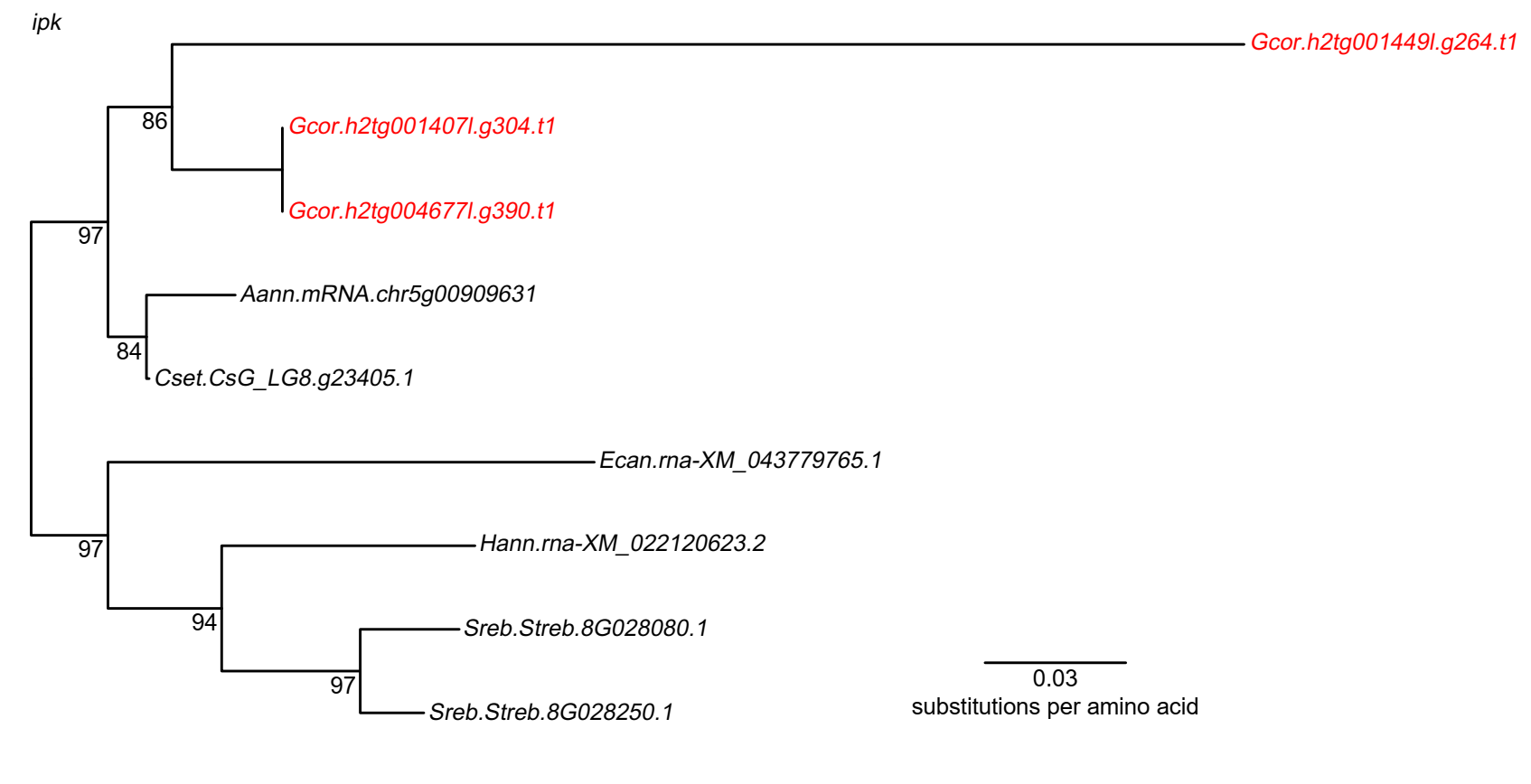


**Figure S13.** Phylogeny tree of Isopentenyl phosphate kinase gene (*ipk*) in 6 Asteroideae species, *Helianthus annuus (Hann)*, *Stevia rebaudiana (Sreb)*, *Erigeron canadensis (Ecan)*, *Glebionis coronaria (Gcor)*, *Artemisia annua (Aann)*, and *Chrysanthemum seticuspe (Cset)*. The tree was constructed using FastTree version 2.1.11^2^ with the multiple protein sequence alignment generated by Muscle version 3.8.31^3^. Branch lengths indicate the phylogenetic distances and integers at splits refer to the number of bootstraps support the corresponding splits.

**Figure S14.** Number of expressed transcripts of monoterpenoid synthesis genes *isopiperitenol dehydrogenase*, *8-hydroxygeraniol dehydrogenase*, and *(S)-8-oxocitronellyl enol synthase* in root, stem and leaf tissue of *G. coronaria*.


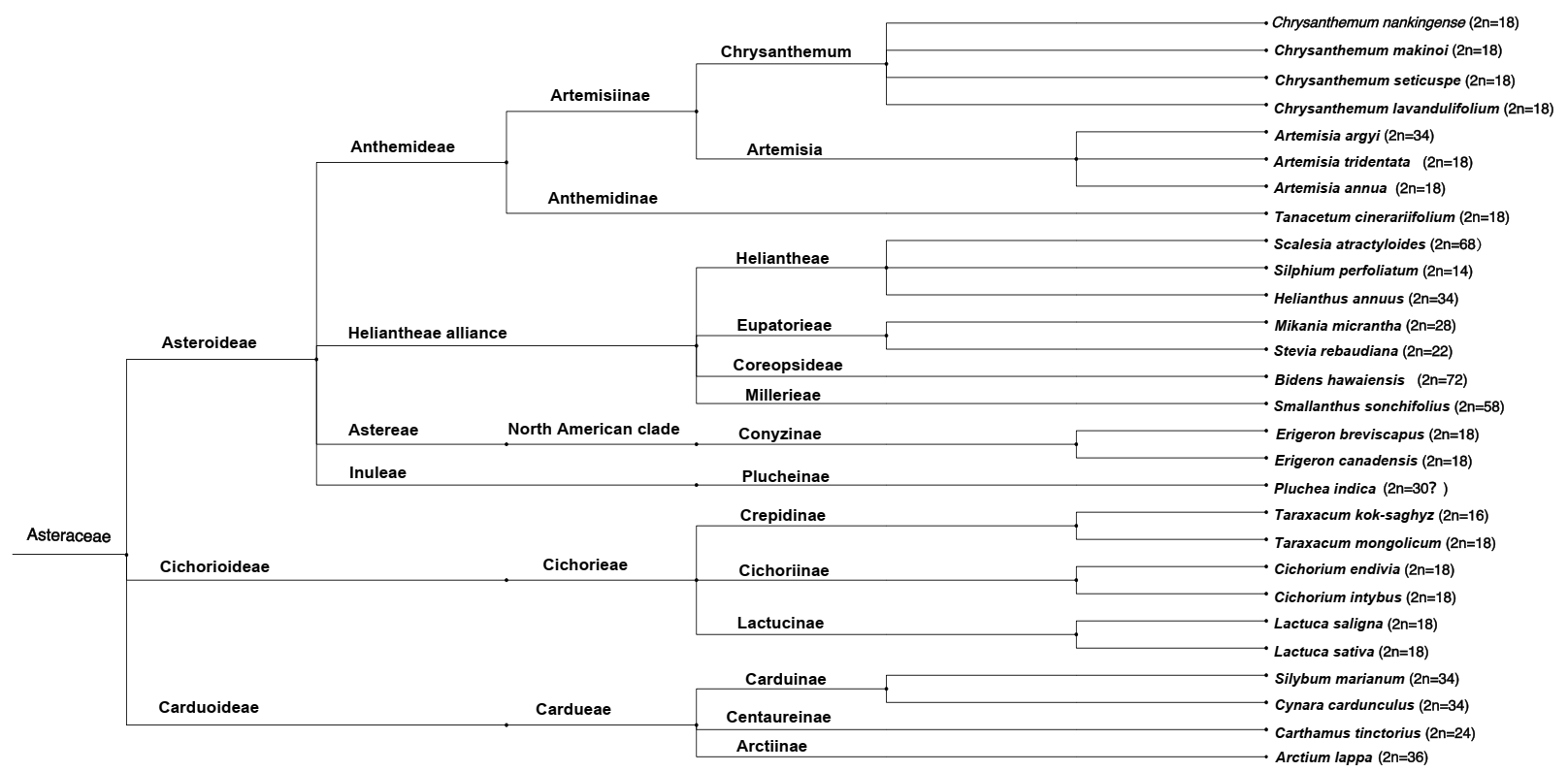


**Figure S15.** Taxonomy of 28 Asteraceae species with published reference genomes^4-34^.

**Supplemental tables**

**Table S1.** Statistics of genome sequencing data of *Glebionis coronaria*

| **Sequencing technology** | **Number of reads** | **Number of bases** | **Coverage of genome** |
| --- | --- | --- | --- |
| PacBio CCS (HiFi) | 12,410,423 | 160,026,900,756 | 23.5 X |
| Hi-C with PE150 | 352,352,819 | 105,684,520,580 | 15.5 X |

**Table S2.** Statistics of mapping Hi-C sequencing reads to assembled contigs of *Glebionis coronaria*

| **Type of read pairs** | **Number of read pairs** | **Percent of read pairs (%)** |
| --- | --- | --- |
| Total pairs processed | 352,352,819 | 100.0% |
| Unmapped pairs | 14,315,341 | 4.1% |
| Low qual pairs | 172,698,860 | 49.0% |
| Pairs with singleton | 44,107,804 | 12.5% |
| Unique paired alignments | 121,230,814 | 34.4% |
| Valid interaction pairs | 51,959,168 | 14.7% |
| Dangling end pairs | 46,221,118 | 13.1% |
| Re-ligation pairs | 22,478,161 | 6.4% |
| Self-cycle pairs | 16,022 | 0.0% |
| Filtered pairs | 538,060 | 0.2% |
| Dumped pairs | 18,285 | 0.0% |
| Valid interaction | 51,959,168 | 14.7% |
| Valid interaction rmdup | 42,843,865 | 12.2% |
| Trans interaction | 22,939,691 | 6.5% |
| Cis interaction | 19,904,174 | 5.6% |

Note: the above statistics were generated by HiC-Pro and the “valid interaction rmdup” read pairs were used by EndHiC for scaffolding.

**Table S3.** Statistics of genome assembly of *Glebionis coronaria*

| **Assembly feature** | **Value** |
| --- | --- |
| Total length of Hifiasm hap1 contigs (bp) | 6,904,706,184 |
| Hap1 contig N50 size (bp) | 3,838,563 |
| BUSCO results of hap1 contigs | C:94.5% [S:88.4%, D:6.1%], F:0.8%, M:4.7% |
| Total length of Hifiasm hap2 contigs (bp) | 6,805,672,885 |
| Hap2 contig N50 size (bp) | 3,862,136 |
| BUSCO results of hap2 contigs | C:94.6% [S:88.2%, D:6.4%], F:1.0%, M:4.4% |
| **Hap2 contigs as reference genome** |  |
| Total length of organelle contigs (bp) | 6,276,267 |
| Total nucleus contig length (bp) | 6,799,396,618 |
| Number of nucleus contigs | 5,135 |
| Contig N50 size (bp) | 3,868,525 |
| Contig N90 size (bp) | 865,646 |
| Total scaffold length (bp) | 6,801,056,618 |
| Number of scaffolds | 3,476 |
| Scaffold N50 size (bp) | 610,719,037 |
| Scaffold N90 size (bp) | 865,646 |
| Total length of chromosome scaffolds (bp) | 5,790,371,166 |
| BUSCO results of eudicots_odb10 | C:94.6% [S:88.2%, D:6.4%], F:1.0%, M:4.4% |

Note: The hap1 and hap2 contig assemblies were generated by Hifiasm (hic mode) with HiFi reads and Hi-C data as input, and the contig set of hap2 with slightly higher BUSCO completeness and contigutiy was selected as reference genome assembly. The organelle contigs were identified by mapping to chloroplast and mitochondrion genomes and removed to get the nucleus contigs. In the BUSCO results, C means complete, S means complete single copy, D means complete duplicate, F means fragmental, and M means missing genes of the 2,326 genes in eudicots_odb10 database.

**Table S4.** Statistics of repeat annotation in the genome of *Glebionis coronaria*

| **Repeat feature** | **Length (bp)** | **N50 size (bp)** | **Percent of length to genome (%)** |
| --- | --- | --- | --- |
| Total TR | 407,425,333 | 226 | 5.9% |
| Intact TE | 1,449,190,144 | 11,864 | 21.3% |
| Homology TE | 4,792,538,624 | 3,808 | 70.4% |
| Denovo TE | 90,323,249 | 523 | 1.3% |
| Total TE | 6,332,052,017 | 5,152 | 93.1% |
| Total 80TE | 6,299,273,163 | 5,196 | 92.6% |

Note: TR is short for tandem repeat, and TE is short for transposon element. Intact TEs were predicted by EDTA based on structural characters of TE, homology TEs were identified by RepeatMasker based on sequence similarity to intact TEs and known TEs in RepBase and TEprotein, and denovo TEs were identified by RepeatMasker and RepeatModeler based on sequence copy number. Total TE refers to all the identified intact TEs, homology TEs and denovo TEs, and Total 80TE refers to all the identified TEs with sizes >= 80 bp.

**Table S5.** Statistics of different transposon elements (TEs) in the genome of *Glebionis coronaria*

| **TE class / order** | **TE superfamily** | **Total length (bp)** | **Percent of length to genome (%)** |
| --- | --- | --- | --- |
| Class I / LTR |  | 5,727,887,039 | 84.2% |
|  | Gypsy | 2,568,861,872 | 37.8% |
|  | Copia | 1,375,390,149 | 20.2% |
|  | LTR | 1,368,959,394 | 20.1% |
|  | Unknown | 410,113,115 | 6.0% |
| Class I / LINE |  | 16,781,832 | 0.3% |
|  | L1 | 13,279,803 | 0.2% |
| Class I / SINE |  | 1,321,328 | 0.0% |
| Class II / DNA |  | 342,837,880 | 5.0% |
|  | Mutator | 130,350,004 | 1.9% |
|  | CMC-EnSpm | 108,104,531 | 1.6% |
|  | hAT | 50,990,816 | 0.7% |
|  | PIF-Harbinger | 31,600,173 | 0.5% |
|  | TcMar-Tc1 | 12,250,460 | 0.2% |
| Class II / MITE |  | 29,899,115 | 0.4% |
| Class II / Helitron |  | 213,320,416 | 3.1% |
| Other |  | 5,289 | 0.0% |

**Table S6.** Statistics of full-length transcriptome sequencing data and alignment of full-length cDNAs to the genome of *Glebionis coronaria*

| **Tissue** | **Type of transcripts** | **Number of transcripts** | **Percent of transcripts (%)** |
| --- | --- | --- | --- |
| Leaf | Transcripts sequenced by Iso-Seq | 69,674 | 100.0 |
|  | Isoseq3 full-length transcripts | 67,895 | 97.4 |
|  | Isoseq3 non-redundant transcripts | 67,135 | 96.4 |
|  | Transcripts aligned to genome | 63,161 | 90.7 |
| Stem | Transcripts sequenced by Iso-Seq | 69,612 | 100.0 |
|  | Isoseq3 full-length transcripts | 67,715 | 97.3 |
|  | Isoseq3 non-redundant transcripts | 61,368 | 88.2 |
|  | Transcripts aligned to genome | 57,373 | 82.4 |
| Root | Transcripts sequenced by Iso-Seq | 45,773 | 100.0 |
|  | Isoseq3 full-length transcripts | 44,671 | 97.6 |
|  | Isoseq3 non-redundant transcripts | 43,802 | 95.7 |
|  | Transcripts aligned to genome | 40,751 | 89.0 |

Note: Iso-Seq is a sequencing technology developed by PacBio to sequence full-length transcripts, and IsoSeq3 is a software package developed by PacBio to process the sequencing data of Iso-Seq and generate full-length and non-redundant transcripts. Gmap and filterPSL.pl (an Augustus script, with parameters ‘--best --minId=95 --minCover=95’) were used to produce the alignments of full-length non-redundant transcripts to genome, and blat2hints.pl (another Augustus script) was used to convert the transcript alignments to the hints file used for gene prediction.

**Table S7.** Number of homology-predicted genes in the genome of *Glebionis coronaria*

| **Species used for homology prediction** | **Number of predicted genes** |
| --- | --- |
| *Artemisia annua* | 40,521 |
| *Chrysanthemum nankingense* | 34,835 |
| *Erigeron canadensis* | 19,746 |
| *Helianthus annuus* | 31,215 |

Note: the protein sequences of the above 4 species were mapped to the genome of *Glebionis coronaria* by Exonerate, and for each query gene, no more than two best target predictions were retained, with the alignment score of the second best should be > 90% of that of the best. These alignments were converted to hints file by Augustus script exonerate2hints.pl.

**Table S8.** Statistics of predicted protein-coding genes in the genome of *Glebionis coronaria*

| **Gene feature** | **Value** |
| --- | --- |
| Number of predicted genes | 104,192 |
| Number of transposon genes | 28,102 |
| Number of protein-coding genes | 76,090 |
| Average CDS length per gene (bp) | 910 |
| Average exon number per gene (bp) | 4.5 |
| Number of genes with hints (%) | 34,718 |
| Percent of genes with hints (%) | 45.6% |
| BUSCO results of eudicots_odb10 | C:94.8% [S:86.8%, D:8.0%], F:1.0%, M:4.2% |

Note: The predicted genes were generated by Augustus on the TE (> 80 bp) soft-masked genome with the hints file of transcript and homology protein alignments, and transposon genes were identified by searching against the NCBI-NR database and removed to get the protein-coding genes. In the BUSCO results, C means complete, S means complete single copy, D means complete duplicate, F means fragmental, and M means missing genes of the 2,326 genes in eudicots_odb10 database.

**Table S9.** Statistics of predicted non-coding RNA genes in the genome of *Glebionis coronaria*

| **Type of non-coding RNA** | **Number of non-coding RNA genes** |
| --- | --- |
| 5S rRNA genes | 706 |
| 18S rRNA genes | 506 |
| 28S rRNA genes | 496 |
| Met tRNA genes | 905 |
| Asp tRNA genes | 828 |
| Asn tRNA genes | 678 |
| Ser tRNA genes | 612 |
| Thr tRNA genes | 607 |
| Ala tRNA genes | 570 |
| Ile tRNA genes | 557 |
| His tRNA genes | 424 |
| Glu tRNA genes | 420 |
| Gly tRNA genes | 344 |
| Val tRNA genes | 331 |
| Arg tRNA genes | 313 |
| Leu tRNA genes | 303 |
| Lys tRNA genes | 277 |
| Pro tRNA genes | 228 |
| Gln tRNA genes | 215 |
| Phe tRNA genes | 207 |
| Cys tRNA genes | 102 |
| Trp tRNA genes | 101 |
| Tyr tRNA genes | 99 |

Note: rRNA and tRNA genes were predicted by tRNAScan-SE version 2.0^35^ and RNAmmer version 1.2^36^, respectively.

**Table S10.** Statistics of function assignment to protein-coding genes of *Glebionis coronaria*

| **Type of genes** | **Number of genes** | **Percent of genes (%)** |
| --- | --- | --- |
| Protein-coding genes | 76,090 | 100.0% |
| Genes with NCBI-NR hits | 57,389 | 75.4% |
| Genes with KEGG hits | 42,241 | 55.5% |
| Genes with InterProScan hits | 61,926 | 81.4% |
| Genes with InterPro terms | 45,163 | 59.4% |
| Genes with GO terms | 32,579 | 42.8% |
| Genes with function terms | 66,063 | 86.8% |

Note: the protein-coding genes were searched against NCBI-NR, KEGG databases using Diamond and InterPro databases using InterProScan to get the functional assignments. Genes with function terms refer to the genes with at least one term in NCBI-NR, KEGG, InterPro, or GO database.

**Table S11.** Summary of public genome data used in this study

| **Species** | **Karyotype** | **Sequencing technology** | **Assembly size** | **Assembly level** | **Scaffold N50 size** | **Data source** | **Data version** |
| --- | --- | --- | --- | --- | --- | --- | --- |
| *Artemisia annua LQ-9* | 2n = 18 | PacBio, Bionano, Hi-C | 1.55 Gb | Chromosome | 71.0 Mb | Global PharmacopoeiaGenome Database | Phase0 |
| *Chrysanthemum nankingense* | 2n = 18 | Illumina, Nanopore | 2.53 Gb | Scaffold | 130.7 Kb | Chrysanthemum Genome Database | V2.0 |
| *Chrysanthemum seticuspe* | 2n = 18 | Illumina, PacBio,  Hi-C | 3.05 Gb | Chromosome | 347.1 Mb | Plant GARDEN | CsGojo-0_v1 |
| *Coffea canephora* | 2n = 22 | Roche 454, Sanger, genetic map | 0.57 Gb | Chromosome | 29.1 Mb | NCBI | AUK_PRJEB4211_v1 |
| *Erigeron canadensis* | 2n = 18 | PacBio,  Hi-C | 0.43 Gb | Chromosome | 45.6 Mb | NCBI | C_canadensis_v1 |
| *Helianthus annuus* | 2n = 34 | PacBio, genetic map, physical map | 3.00 Gb | Chromosome | 176.5 Mb | NCBI | HanXRQr2.0-SUNRISE |
| *Lactuca sativa* | 2n = 18 | Illumina, Hi-C, genetic map | 2.38 Gb | Chromosome | 257.9 Mb | NCBI | Lsat_Salinax_v7 |
| *Smallanthus sonchifolius* | 2n = 58 | PacBio,  Hi-C | 2.72 Gb | Chromosome | 91.5 Mb | NCBI | ASM2352597v1 |
| *Stevia rebaudiana* | 2n = 22 | PacBio,  Hi-C | 1.41 Gb | Chromosome | 106.6 Mb | FigShare | 14169491.v1 |

Note: Assembly size and Scaffold N50 size are calculated from the downloaded genome sequences by in-house scripts.

**Table S12.** Statistics of public gene sets used in this study

| **Species** | **Number of genes** | **Total CDS length (bp)** | **Percent of total CDS to genome (%)** | **Average CDS length per gene (bp)** | **Average exon number per gene** |
| --- | --- | --- | --- | --- | --- |
| *Artemisia annua LQ-9* | 54,347 | 52,988,195 | 3.4% | 975 | 4.3 |
| *Chrysanthemum nankingense* | 56,870 | 57,335,697 | 2.3% | 1,008 | 4.6 |
| *Chrysanthemum seticuspe* | 74,259 | 103,156,615 | 3.4% | 1,389 | 5.4 |
| *Coffea canephora* | 25,574 | 30,830,841 | 5.4% | 1,206 | 5.1 |
| *Erigeron canadensis* | 28,703 | 38,515,296 | 9.1% | 1,342 | 5.2 |
| *Helianthus annuus* | 57,126 | 71,300,168 | 2.4% | 1,248 | 4.3 |
| *Lactuca sativa* | 36,136 | 45,233,032 | 1.9% | 1,252 | 4.7 |
| *Smallanthus sonchifolius* | 89,960 | 98,187,351 | 3.6% | 1,092 | 5.1 |
| *Stevia rebaudiana* | 44,143 | 53,584,410 | 3.8% | 1,214 | 5.0 |

Note: The CDS sequences of genes were extracted from the downloaded genome sequences (FASTA) and the corresponding gene annotation files (GFF3), and translated into protein sequences using in-house scripts. For genes with multiple transcripts, the longest transcript was chosen as the representative.

**Table S13.** Summary of the type of duplicate genes in *Glebionis coronaria*

| **Type** | **Number of genes** | **Percent of genes (%)** |
| --- | --- | --- |
| Singleton | 9,019 | 14.2% |
| Dispersed | 33,036 | 51.9% |
| Proximal | 7,180 | 11.3% |
| Tandem | 9,804 | 15.4% |
| WGD / Segmental | 4,570 | 7.2% |
| Total | 63,069 | 100.0% |

Note: the above results were obtained by running the duplicate_gene_classifier of MCScanX software with the all-vs-all alignment of protein-coding genes anchored to chromosomes as input. Singleton means single-copy genes, Dispersed means genes with duplicates distributed randomly in genome, Proximal means genes with duplicates located nearby each other, Tandem means genes with exact tandem duplicates, WGD / Segmental means genes with duplicates located on long colinear fragments.

**Table S14.** Copy number of genes involved in terpenoid backbone biosynthesis.

| **Gene** | **Full name** | ***Aann*** | ***Cset*** | ***Ecan*** | ***Gcor*** | ***Hann*** | ***Sreb*** |
| --- | --- | --- | --- | --- | --- | --- | --- |
| MVA pathway | mevalonate pathway |  |  |  |  |  |  |
| *ACAT* | *acetyl-CoA C-acetyltransferase* | 4 | 3 | 3 | 3 | 2 | 3 |
| *HMGCS* | *hydroxymethylglutaryl-CoA synthase* | 2 | 1 | 2 | 3 | 1 | 2 |
| *HMGCR* | *hydroxymethylglutaryl-CoA reductase* | 4 | 11 | 5 | 7 | 9 | 9 |
| *MVK* | *mevalonate kinase* | 2 | 2 | 1 | 3 | 1 | 1 |
| *PMVK* | *phosphomevalonate kinase* | 3 | 2 | 2 | 2 | 2 | 3 |
| *MVD* | *diphosphomevalonate decarboxylase* | 1 |  | 1 | 1 | 1 | 2 |
| MEP/DOXP pathway | methylerythritol phosphate /  deoxy-xylulose phosphate pathway |  |  |  |  |  |  |
| *dxs* | *1-deoxy-D-xylulose-5-phosphate synthase* | 1 | 1 | 1 | 1 | 1 | 1 |
| *dxr* | *1-deoxy-D-xylulose-5-phosphate reductoisomerase* | 3 | 2 | 1 | 3 | 3 | 2 |
| *ispD* | *2-C-methyl-D-erythritol 4-phosphate cytidylyltransferase* |  | 1 | 1 | 3 | 3 | 3 |
| *ispE* | *4-diphosphocytidyl-2-C-methyl-D-erythritol kinase* | 2 | 1 | 1 | 1 | 1 | 1 |
| *ispF* | *2-C-methyl-D-erythritol 2,4-cyclodiphosphate synthase* | 1 | 1 | 1 | 1 | 1 | 1 |
| *gcpE* | *(E)-4-hydroxy-3-methylbut-2-enyl-diphosphate synthase* | 1 | 3 | 1 | 1 | 5 | 1 |
| *ispH* | *4-hydroxy-3-methylbut-2-en-1-yl diphosphate reductase* | 5 | 4 | 2 | 6 | 4 | 3 |
| Other |  |  |  |  |  |  |  |
| *ipk* | *isopentenyl phosphate kinase* | 1 | 1 | 1 | 3 | 1 | 2 |
| *idi* | *isopentenyl-diphosphate Delta-isomerase* | 2 | 3 | 2 | 2 | 2 | 2 |
| *ispS* | *isoprene synthase* | 6 | 10 | 3 | 4 | 3 | 11 |
| *GGPS* | *geranylgeranyl diphosphate synthase* | 1 | 2 | 1 | 1 | 3 | 1 |
| *FLDH* | *NAD+-dependent farnesol dehydrogenase* | 3 | 3 | 1 | 6 | 1 | 1 |
| *FOLK* | *farnesol kinase* | 2 | 3 | 3 | 5 | 3 | 4 |
| *chlP* | *geranylgeranyl diphosphate* | 3 | 4 | 2 | 3 | 4 | 2 |
| *SPS* | *all-trans-nonaprenyl-diphosphate synthase* | 1 | 1 | 1 | 1 | 2 | 2 |
| *PCME* | *prenylcysteine alpha-carboxyl methylesterase* | 2 | 1 | 1 | 1 | 1 | 2 |
| *ICMT* | *protein-S-isoprenylcysteine O-methyltransferase* | 2 | 2 | 1 | 1 | 2 | 3 |
| *STE24* | *STE24 endopeptidase* | 1 | 1 | 1 | 1 | 3 | 1 |
| *FACE2* | *prenyl protein peptidase* | 1 | 1 | 1 | 1 | 6 | 1 |
| *FNTA* | *geranylgeranyltransferase type-1 subunit alpha* | 1 | 1 | 1 | 1 | 1 | 1 |
| *DHDDS* | *ditrans,polycis-polyprenyl diphosphate synthase* | 0 | 0 | 0 | 0 | 2 | 0 |

Note: the homolog genes in each species involved in terpenoid backbone biosynthesis were identified by aligning the predicted protein-coding genes to the known terpenoid backbone biosynthesis genes in KEGG database using Diamond version 0.8.28, and the existence of N-terminal domain pfam01397 and metal binding domain pfam03936 of terpenoid synthases using HMMER version 3.1b2^38^. Then the best hits with identity >= 60% and coverage >= 80% were retained for statistics. Species name abbreviation: *Aann*, *Artemisia annua*; *Cset*, *Chrysanthemum seticuspe*; *Ecan*, *Erigeron canadensis*; *Gcor*, *Glebionis coronaria*; *Hann*, *Helianthus annuus*; *Lsat*, *Lactuca sativa*; *Sreb*, *Stevia rebaudiana*. The genes expanded in *Glebionis coronaria* were highlighted in red.

**Table S15.** Copy number of genes involved in monoterpenoid biosynthesis.

| **Gene** | **Full name** | ***Aann*** | ***Cset*** | ***Ecan*** | ***Gcor*** | ***Hann*** | ***Sreb*** |
| --- | --- | --- | --- | --- | --- | --- | --- |
| *NUDX1* | *geranyl diphosphate phosphohydrolase* | 4 | 2 | 2 | 2 | 2 | 5 |
| *GES* | *geranyl diphosphate diphosphatase* | 1 | 2 | 5 | 0 | 2 | 4 |
| *CYP76B6* | *geraniol 8-hydroxylase* | 12 | 12 | 7 | 3 | 10 | 7 |
| *10HGO* | *8-hydroxygeraniol dehydrogenase* | 16 | 20 | 14 | 20 | 17 | 15 |
| *CrISY* | *(S)-8-oxocitronellyl enol synthase* | 4 | 5 | 5 | 7 | 3 | 2 |
| *NEPS1* | *nepetalactol dehydrogenase / (+)-cis,trans-nepetalactol synthase* | 4 | 6 | 2 | 3 | 1 | 1 |
| *CYP76A26* | *nepetalactol monooxygenase* | 1 | 0 | 0 | 0 | 0 | 0 |
| *UGT8* | *7-deoxyloganetic acid glucosyltransferase* | 5 | 8 | 6 | 3 | 3 | 5 |
| *UGT85A23_24* | *7-deoxyloganetin glucosyltransferase* | 28 | 48 | 17 | 34 | 27 | 26 |
| *CYP72A224* | *7-deoxyloganate 7-hydroxylase* | 18 | 9 | 9 | 14 | 18 | 24 |
| *CYP72A1* | *secologanin synthase* | 9 | 6 | 2 | 7 | 2 | 3 |
| *TPS14* | *(3S)-linalool synthase* | 4 | 1 | 2 | 2 | 4 | 0 |
| *CYP71D13_15_95* | *(S)-limonene 3-monooxygenase* | 1 | 0 | 0 | 0 | 0 | 0 |
| *CYP71D18* | *(S)-limonene 6-monooxygenase* | 3 | 3 | 0 | 0 | 0 | 0 |
| *E1.1.1.223* | *isopiperitenol dehydrogenase* | 2 | 1 | 1 | 5 | 0 | 0 |
| *E1.3.1.82* | *(-)-isopiperitenone reductase* | 1 | 0 | 0 | 0 | 1 | 1 |
| *E1.3.1.81* | *(+)-pulegone reductase* | 15 | 14 | 10 | 13 | 12 | 5 |
| *E1.14.14.143* | *(+)-menthofuran synthase* | 4 | 4 | 1 | 4 | 7 | 3 |
| *E1.1.1.207* | *(-)-**menthol dehydrogenase* | 1 | 0 | 2 | 1 | 0 | 2 |
| *E1.1.1.208* | *(+)-neomenthol dehydrogenase* | 17 | 14 | 7 | 6 | 6 | 14 |
| *AG3* | *(-)-beta-pinene synthase* | 12 | 21 | 2 | 7 | 5 | 4 |

Note: the homolog genes in each species involved in monoterpenoid biosynthesis were identified by aligning the predicted protein-coding genes to the known monoterpenoid biosynthesis genes in KEGG database using Diamond version 0.8.28, and the existence of N-terminal domain pfam01397 and metal binding domain pfam03936 of terpenoid synthases using HMMER version 3.1b2^38^. Then the best hits with identity >= 70% and coverage >= 80% were retained for statistics. Species name abbreviation: *Aann*, *Artemisia annua*; *Cset*, *Chrysanthemum seticuspe*; *Ecan*, *Erigeron canadensis*; *Gcor*, *Glebionis coronaria*; *Hann*, *Helianthus annuus*; *Lsat*, *Lactuca sativa*; *Sreb*, *Stevia rebaudiana*. The genes expanded in *Glebionis coronaria* were highlighted in red.

**References**

1. Gu, Z., Gu, L., Eils, R., Schlesner, M. and Brors, B. 2014, circlize Implements and enhances circular visualization in R. *Bioinformatics*, **30**, 2811-2812.

2. Price, M. N., Dehal, P. S. and Arkin, A. P. 2010, FastTree 2-Approximately Maximum-Likelihood Trees for Large Alignments. *Plos One*, **5**.

3. Edgar, R. C. 2004, MUSCLE: multiple sequence alignment with high accuracy and high throughput. *Nucleic acids research*, **32**, 1792-1797.

4. Zhang, B., Wang, Z., Han, X., et al. 2022, The chromosome-scale assembly of endive (Cichorium endivia) genome provides insights into the sesquiterpenoid biosynthesis. *Genomics*, **114**, 110400.

5. Wen, X., Li, J., Wang, L., et al. 2022, The chrysanthemum lavandulifolium genome and the molecular mechanism underlying diverse capitulum types. *Hortic Res*, **9**.

6. Miao, Y., Luo, D., Zhao, T., et al. 2022, Genome sequencing reveals chromosome fusion and extensive expansion of genes related to secondary metabolism in Artemisia argyi. *Plant Biotechnology Journal*, **n/a**.

7. Melton, A. E., Child, A. W., Beard, R. S., Jr, et al. 2022, A haploid pseudo-chromosome genome assembly for a keystone sagebrush species of western North American rangelands. *G3 Genes|Genomes|Genetics*, **12**.

8. Lin, T., Xu, X., Du, H., et al. 2022, Extensive sequence divergence between the reference genomes of Taraxacum kok-saghyz and Taraxacum mongolicum. *Science China. Life sciences*, **65**, 515-528.

9. Liao, B., Shen, X., Xiang, L., et al. 2022, Allele-aware chromosome-level genome assembly of Artemisia annua reveals the correlation between ADS expansion and artemisinin yield. *Molecular Plant*.

10. He, Z., Feng, X., Chen, Q., et al. 2022, Evolution of coastal forests based on a full set of mangrove genomes. *Nature Ecology & Evolution*, **6**, 738-749.

11. Fan, W., Wang, S., Wang, H., et al. 2022, The genomes of chicory, endive, great burdock and yacon provide insights into Asteraceae palaeo-polyploidization history and plant inulin production. *Molecular Ecology Resources*, **n/a**.

12. Cerca, J., Petersen, B., Lazaro-Guevara, J. M., et al. 2022, The genomic basis of the plant island syndrome in Darwin’s giant daisies. *Nature communications*, **13**, 3729.

13. Bellinger, M. R., Datlof, E., Selph, K. E., Gallaher, T. J. and Knope, M. L. 2022, A genome for Bidens hawaiensis: a member of a hexaploid Hawaiian plant adaptive radiation. *The Journal of heredity*.

14. Yang, Y., Li, S., Xing, Y., et al. 2021, The first high-quality chromosomal genome assembly of a medicinal and edible plant Arctium lappa. *Mol Ecol Resour*.

15. Xu, X., Yuan, H., Yu, X., et al. 2021, The chromosome-level Stevia genome provides insights into steviol glycoside biosynthesis. *Horticulture Research*, **8**, 129.

16. Wu, Z., Liu, H., Zhan, W., et al. 2021, The chromosome-scale reference genome of safflower (Carthamus tinctorius) provides insights into linoleic acid and flavonoid biosynthesis. *Plant Biotechnology Journal*, **n/a**.

17. van Lieshout, N., van Kaauwen, M., Kodde, L., et al. 2021, De novo whole-genome assembly of Chrysanthemum makinoi, a key wild chrysanthemum. *G3 Genes|Genomes|Genetics*, **12**.

18. Nakano, M., Hirakawa, H., Fukai, E., et al. 2021, A chromosome-level genome sequence of Chrysanthemum seticuspe, a model species for hexaploid cultivated chrysanthemum. *Communications Biology*, **4**, 1167.

19. He, S., Dong, X., Zhang, G., et al. 2021, High quality genome of Erigeron breviscapus provides a reference for herbal plants in Asteraceae. *Molecular Ecology Resources*, **21**, 153-169.

20. Liu, B., Yan, J., Li, W. H., et al. 2020, Mikania micrantha genome provides insights into the molecular mechanism of rapid growth. *Nature communications*, **11**, 13.

21. Laforest, M., Martin, S. L., Bisaillon, K., Soufiane, B., Meloche, S. and Page, E. 2020, A chromosome-scale draft sequence of the Canada fleabane genome. *Pest Manag Sci*, **76**, 2158-2169.

22. Acquadro, A., Portis, E., Valentino, D., Barchi, L. and Lanteri, S. 2020, “Mind the Gap”: Hi-C Technology Boosts Contiguity of the Globe Artichoke Genome in Low-Recombination Regions. *G3: Genes|Genomes|Genetics*, **10**, 3557-3564.

23. Song, C., Liu, Y., Song, A., et al. 2018, The Chrysanthemum nankingense Genome Provides Insights into the Evolution and Diversification of Chrysanthemum Flowers and Medicinal Traits. *Molecular Plant*, **11**, 1482-1491.

24. Reyes-Chin-Wo, S., Wang, Z., Yang, X., et al. 2017, Genome assembly with in vitro proximity ligation data and whole-genome triplication in lettuce. *Nature communications*, **8**, 14953.

25. Badouin, H., Gouzy, J., Grassa, C. J., et al. 2017, The sunflower genome provides insights into oil metabolism, flowering and Asterid evolution. *Nature*, **546**, 148-152.

26. Melton, A. E., Beck, J., Galla, S. J., et al. 2021, A draft genome provides hypotheses on drought tolerance in a keystone plant species in Western North America threatened by climate change. *Ecology and evolution*, **11**, 15417-15429.

27. Yamashiro, T., Shiraishi, A., Satake, H. and Nakayama, K. 2019, Draft genome of Tanacetum cinerariifolium, the natural source of mosquito coil. *Scientific Reports*, **9**, 18249.

28. Hirakawa, H., Sumitomo, K., Hisamatsu, T., et al. 2019, De novo whole-genome assembly in Chrysanthemum seticuspe, a model species of Chrysanthemums, and its application to genetic and gene discovery analysis. *DNA Res.*, **26**, 195-203.

29. Verwaaijen, B., Wibberg, D., Nelkner, J., et al. 2018, Assembly of the Lactuca sativa, L. cv. Tizian draft genome sequence reveals differences within major resistance complex 1 as compared to the cv. Salinas reference genome. *J. Biotechnol.*, **267**, 12-18.

30. Shen, Q., Zhang, L. D., Liao, Z. H., et al. 2018, The Genome of Artemisia annua Provides Insight into the Evolution of Asteraceae Family and Artemisinin Biosynthesis. *Molecular Plant*, **11**, 776-788.

31. Yang, J., Zhang, G., Zhang, J., et al. 2017, Hybrid de novo genome assembly of the Chinese herbal fleabane Erigeron breviscapus. *GigaScience*, **6**.

32. Lin, T., Xu, X., Ruan, J., et al. 2017, Genome analysis of Taraxacum kok-saghyz Rodin provides new insights into rubber biosynthesis. *National Science Review*, **5**, 78-87.

33. Scaglione, D., Reyes-Chin-Wo, S., Acquadro, A., et al. 2016, The genome sequence of the outbreeding globe artichoke constructed de novo incorporating a phase-aware low-pass sequencing strategy of F1 progeny. *Scientific Reports*, **6**, 19427.

34. Peng, Y., Lai, Z., Lane, T., et al. 2014, De Novo Genome Assembly of the Economically Important Weed Horseweed Using Integrated Data from Multiple Sequencing Platforms. *Plant Physiology*, **166**, 1241-1254.

35. Lowe, T. M. and Chan, P. P. 2016, tRNAscan-SE On-line: integrating search and context for analysis of transfer RNA genes. *Nucleic acids research*, **44**, W54-W57.

36. Lagesen, K., Hallin, P., Rødland, E. A., Staerfeldt, H. H., Rognes, T. and Ussery, D. W. 2007, RNAmmer: consistent and rapid annotation of ribosomal RNA genes. *Nucleic acids research*, **35**, 3100-3108.

37. Jin, J., Tian, F., Yang, D.-C., et al. 2016, PlantTFDB 4.0: toward a central hub for transcription factors and regulatory interactions in plants. *Nucleic acids research*, **45**, D1040-D1045.

38. Potter, S. C., Luciani, A., Eddy, S. R., Park, Y., Lopez, R. and Finn, R. D. 2018, HMMER web server: 2018 update. *Nucleic acids research*, **46**, W200-W204.
